# Supplementary figures and images for: The multiscale brain structural re-organization that occurs from childhood to adolescence correlates with cortical morphology maturation and functional specialization
Source: PLoS Biol. 2025 Apr 1;23(4):e3002710. doi: 10.1371/journal.pbio.3002710 (PMC12017512; doi:10.1371/journal.pbio.3002710)

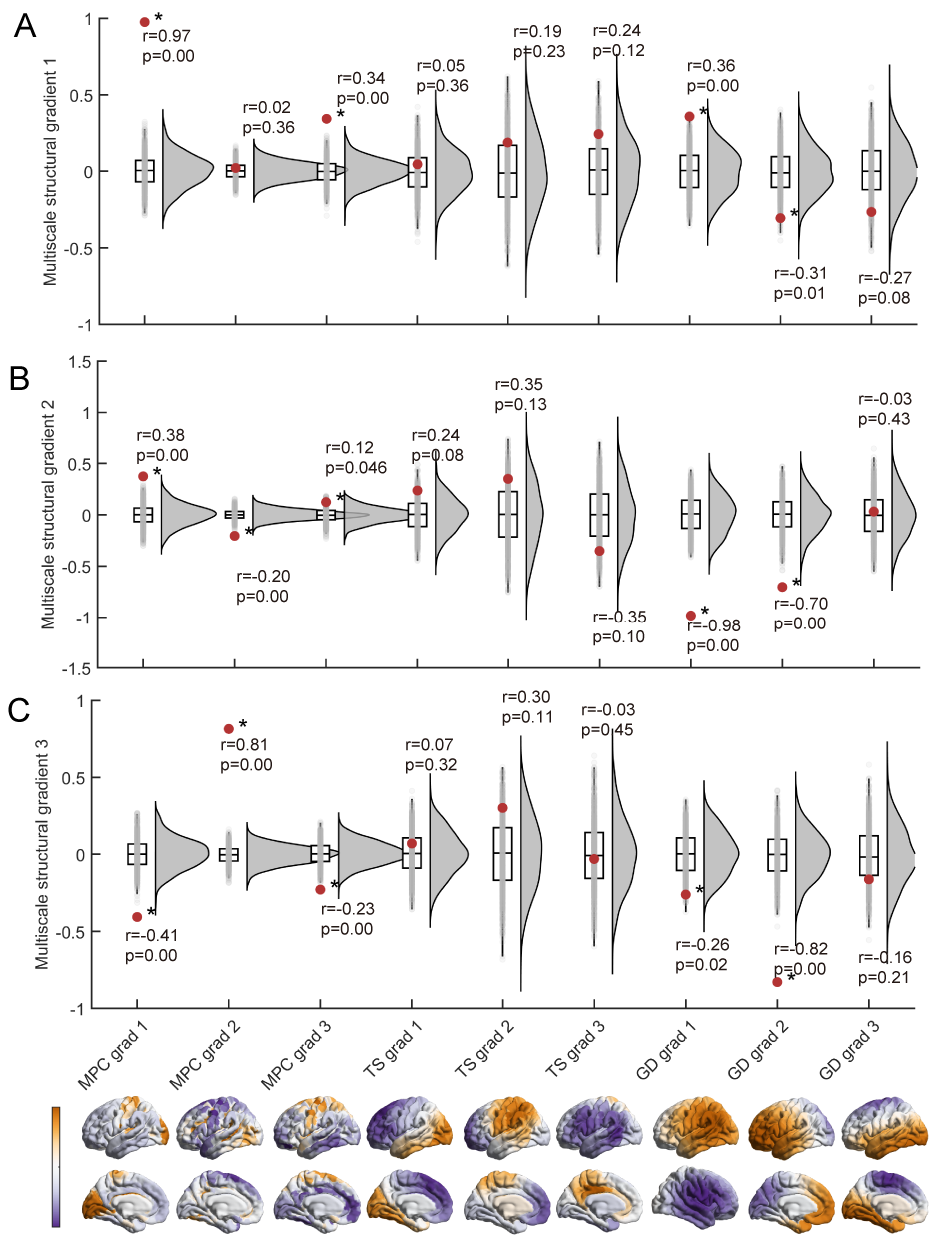

Supplement: S1 Fig — The x-axis represents the top three gradients of MPC, TS, and GD, while the y-axis represents the partial correlations with multiscale structural gradients. The top, middle, and bottom plots correspond to the top three multiscale structural gradients, respectively. Red dots indicate actual correlation coefficients. Spatial autocorrelation was corrected using 1,000 surrogate maps generated via the variogram matching approach [54]. Box plots and density plots show the null distribution of 1,000 correlation coefficients. The data underlying this figure can be found at https://zenodo.org/records/14874537. (TIF) [file pbio.3002710.s001.tif]

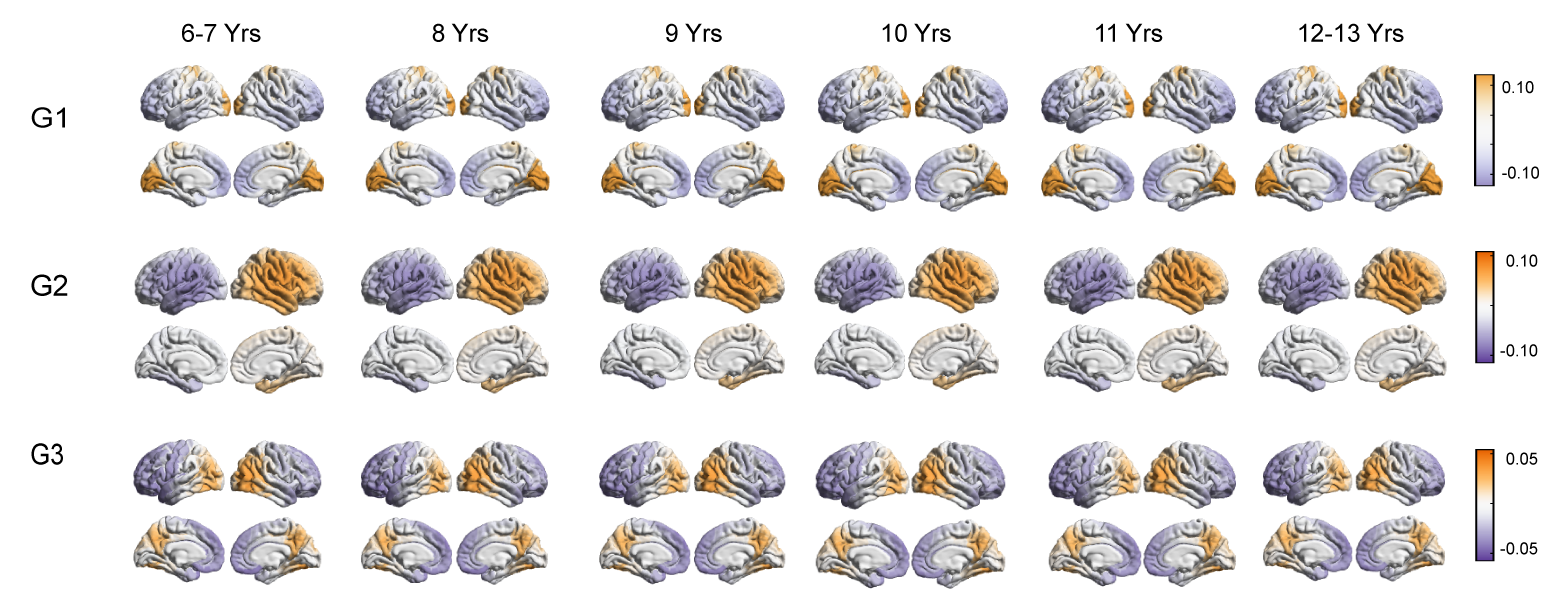

Supplement: S2 Fig — (TIF) [file pbio.3002710.s002.tif]

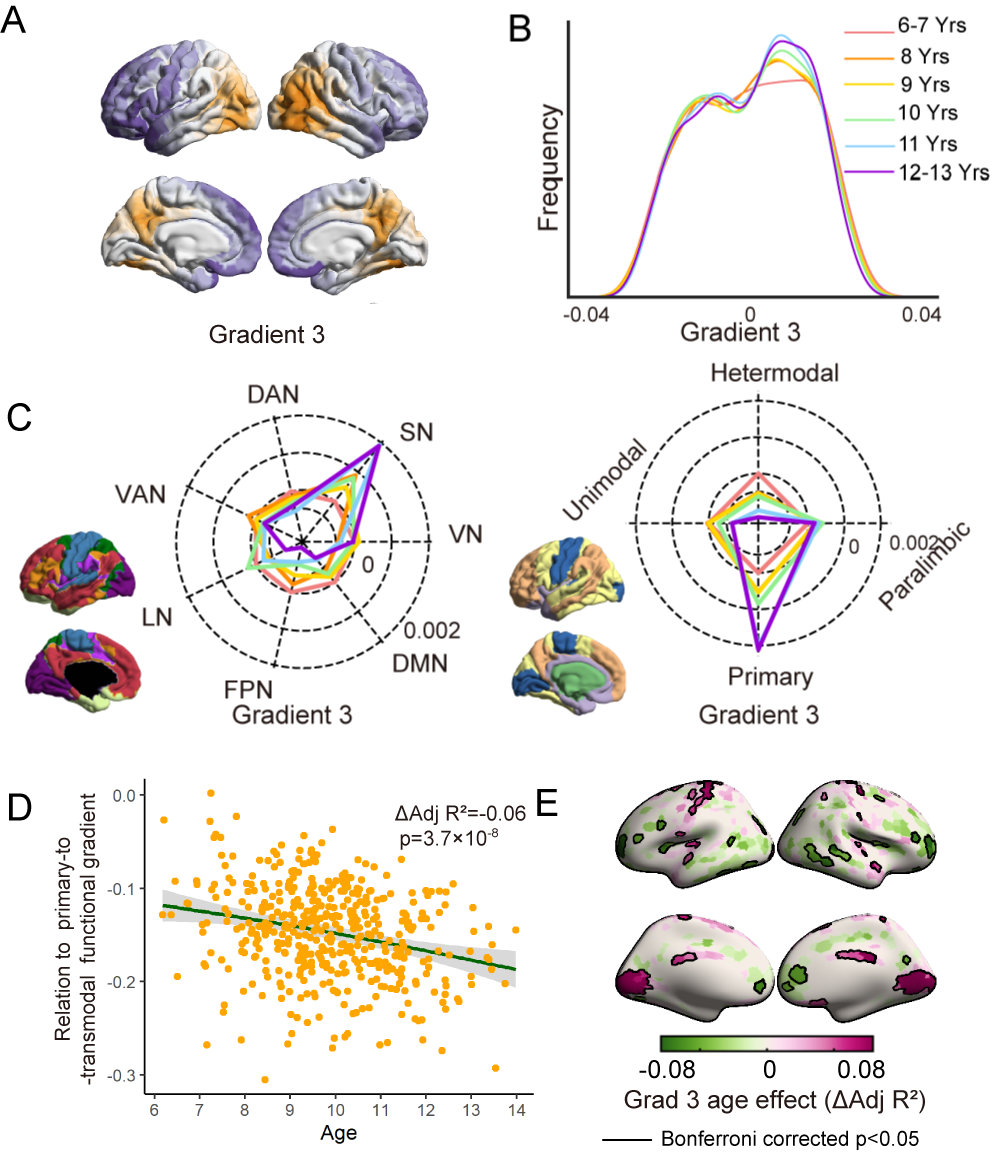

Supplement: S3 Fig — (A) The third gradient on the cortical surface. (B) Global density map of the third gradient for each group. (C) Radar plot of the third gradient for comparison between 6–7 years group and other groups based on Yeo functional networks (left) [47] and laminar differentiation parcellation (right) [48]. (D) Correlation coefficient between the third structural gradient and primary-to-transmodal functional gradient changed across age. (E) The spatial pattern of age-effect on the third gradient, with the age effect quantified using ΔAdj R2. The results that survived Bonferroni correction are circled by black lines (Bonferroni corrected p < 0.05). The data underlying this figure can be found at https://zenodo.org/records/14874537. (TIF) [file pbio.3002710.s003.tif]

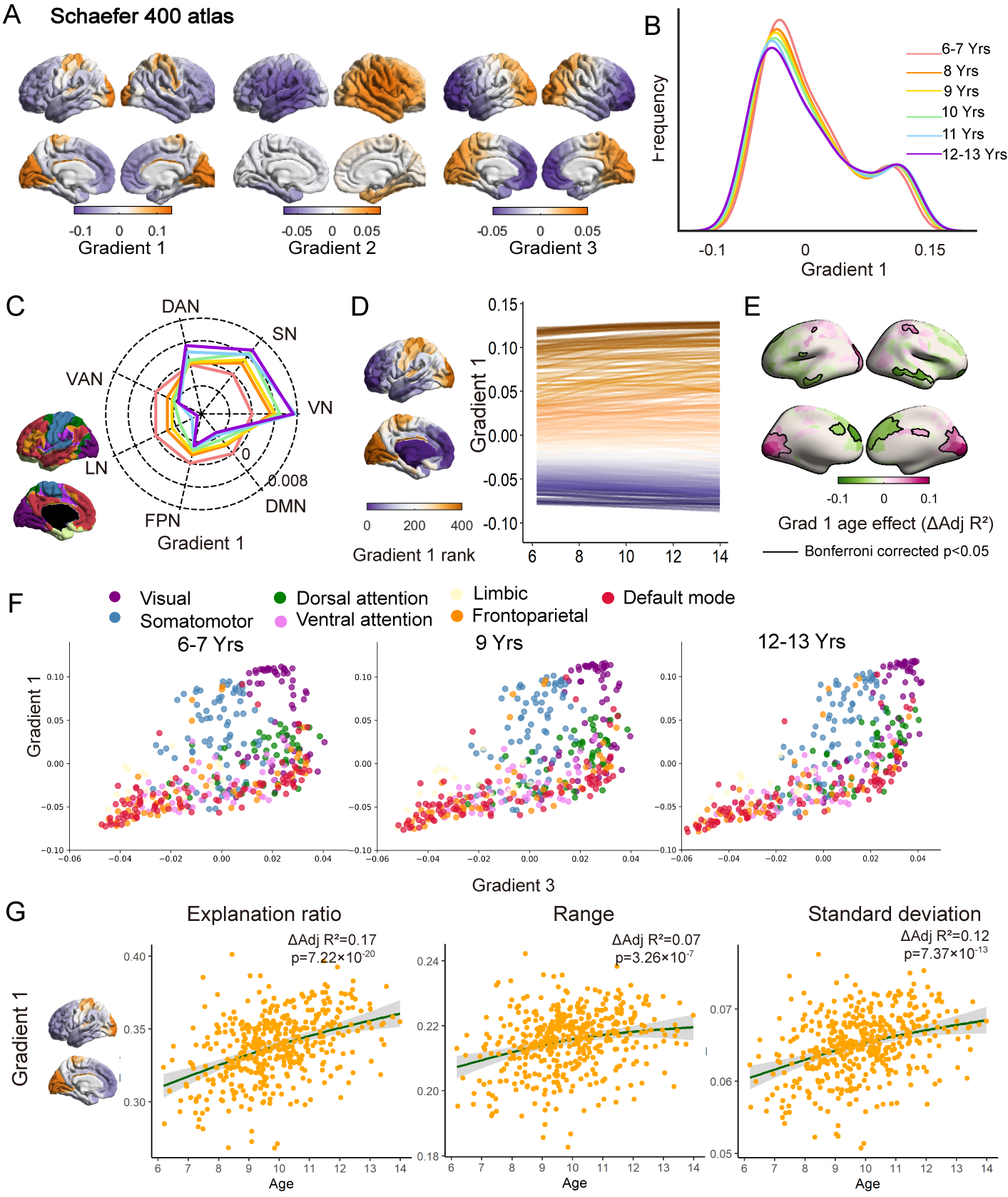

Supplement: S4 Fig — (A) The group-level gradients based on the Schaefer 400 atlas. (B) Global density map of the principal gradient for each group. (C) Radar plot of the principal gradient for comparison between 6–7 years group and other groups based on Yeo functional networks [47]. (D) The development trajectory of gradient scores for each region. (E) The spatial pattern of age-effect on the principal gradient, with the age effect quantified using ΔAdj R2. The results that survived Bonferroni correction are circled by black lines (Bonferroni corrected p < 0.05). (F) The first and third structural gradients mapped into a 2D gradient space for 6–7, 9, and 12–13 years old group demonstrated an expansion pattern during development. (G) Global measures of the principal gradient changed across age groups, including the explanation ratio (left), range (middle), and standard deviation (right) (age-effect p < 0.01). (TIF) [file pbio.3002710.s004.tif]

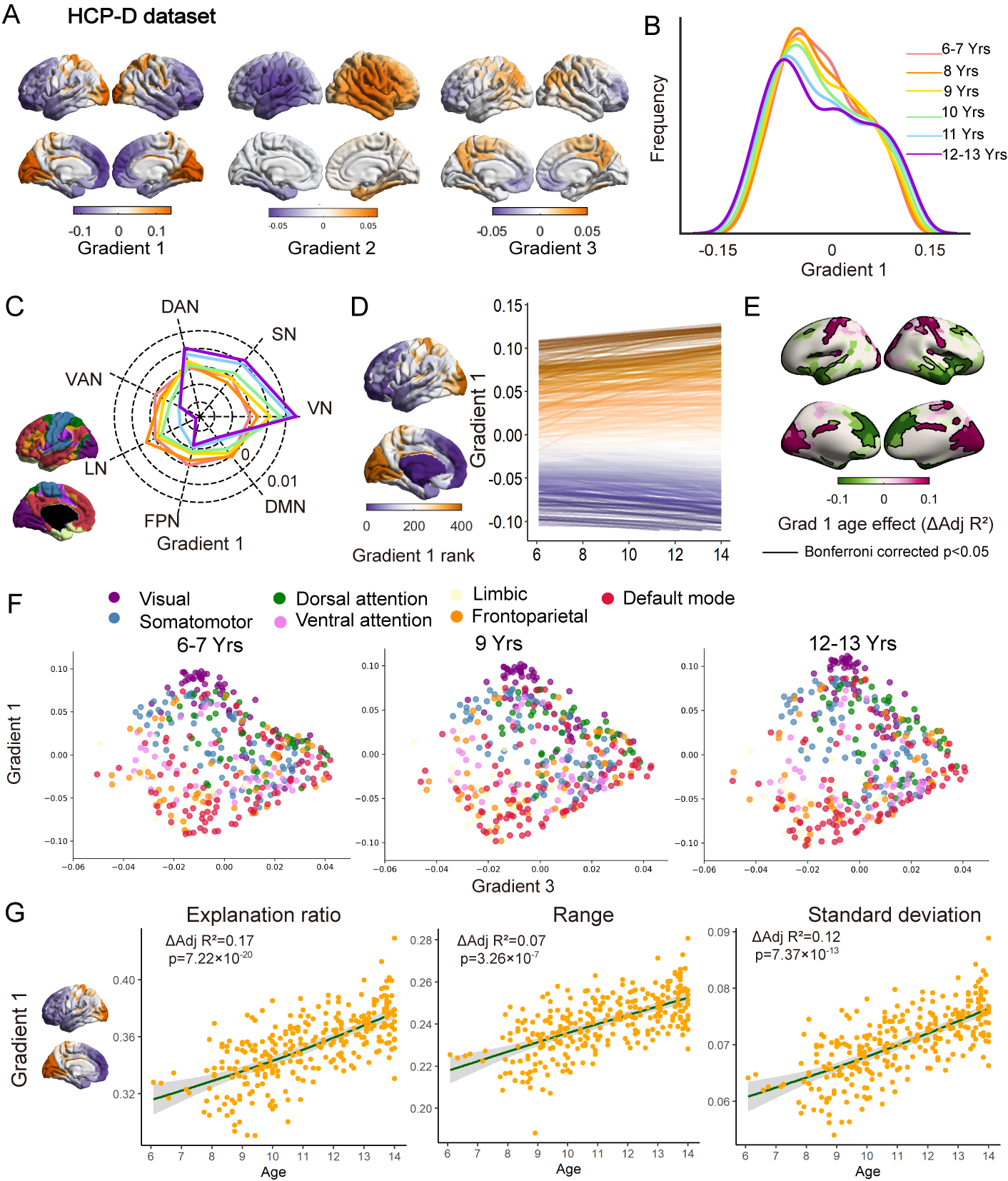

Supplement: S5 Fig — The same steps were conducted to regenerate the multiscale structural gradients using an independent HCP-D dataset aged 6–14 years old. (TIF) [file pbio.3002710.s005.tif]

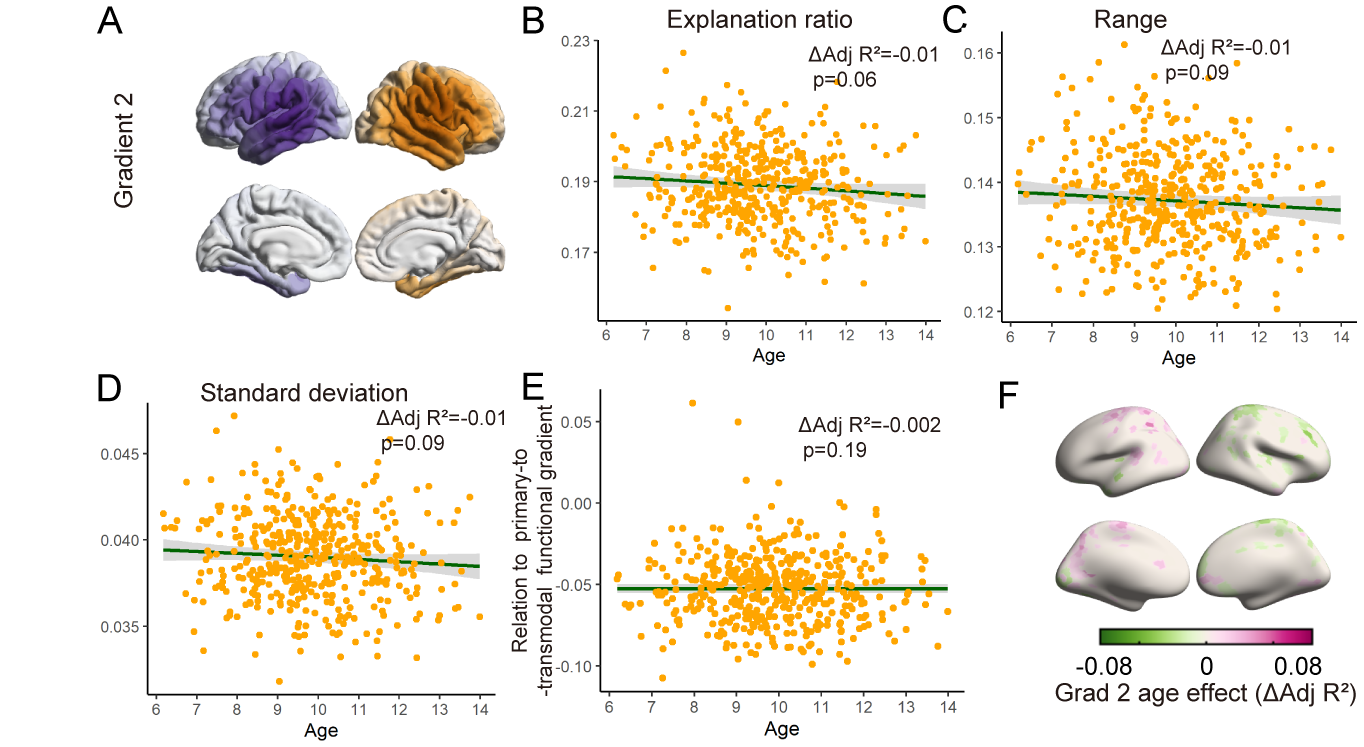

Supplement: S6 Fig — (A) The second gradient on the cortical surface. (B–D) Global measures of the principal gradient changed across age groups, including the explanation ratio (B), range (C), and standard deviation (D) (age-effect are not significant). (E) Correlation coefficient between the second structural gradient and primary-to-transmodal functional gradient changed across age. (F) The spatial pattern of age-effect on the second gradient, with the age effect quantified using ΔAdj R2. (TIF) [file pbio.3002710.s006.tif]

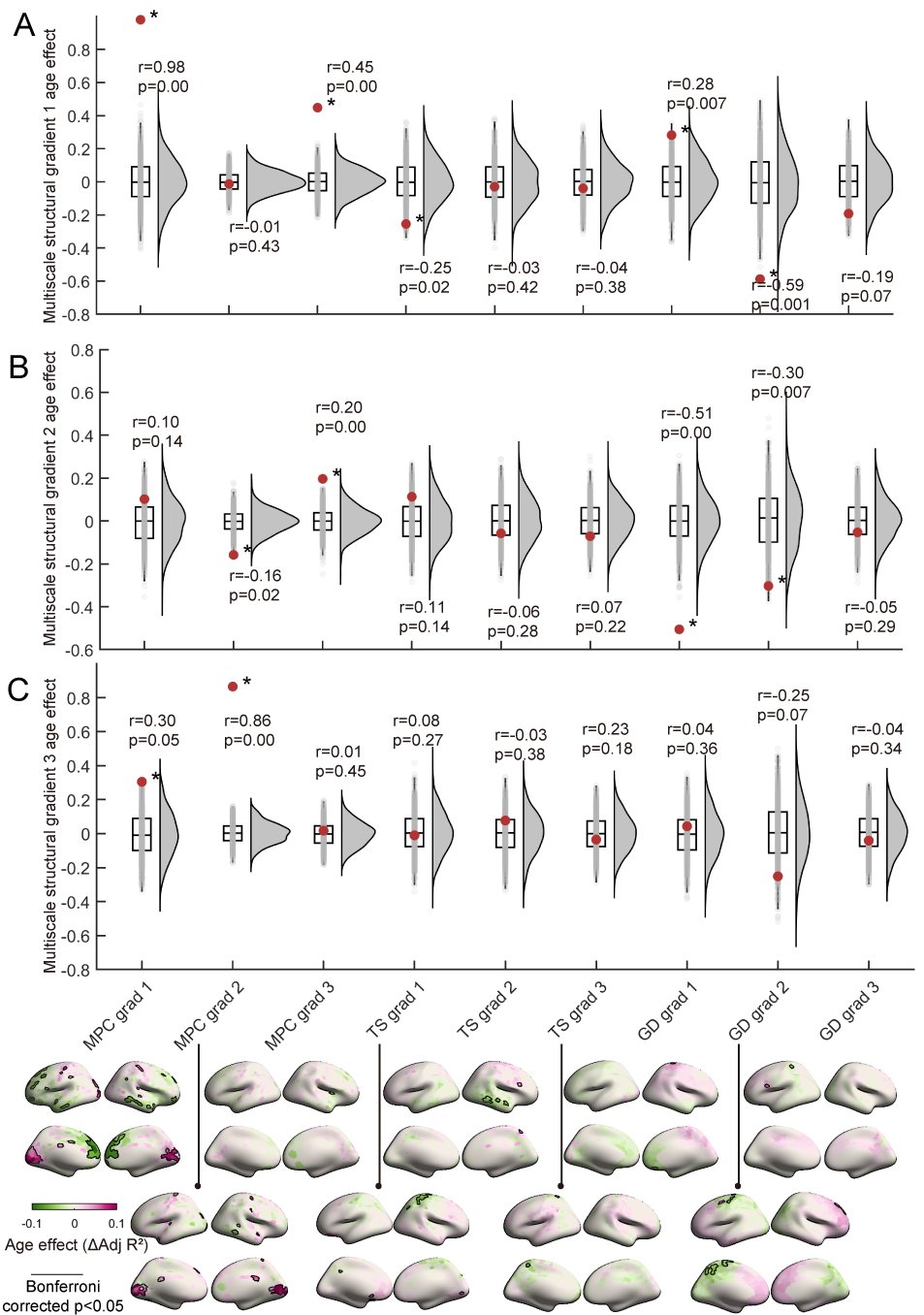

Supplement: S7 Fig — The x-axis represents the developmental effects of top three gradients of MPC, TS, and GD, while the y-axis represents the developmental effects of multiscale structural gradients. The top, middle, and bottom plots correspond to the top three multiscale structural gradients, respectively. Red dots indicate actual correlation coefficients. Spatial autocorrelation was corrected using 1,000 surrogate maps generated via the variogram matching approach. Box plots and density plots show the null distribution of 1,000 correlation coefficients. The data underlying this figure can be found at https://zenodo.org/records/14874537. (TIF) [file pbio.3002710.s007.tif]

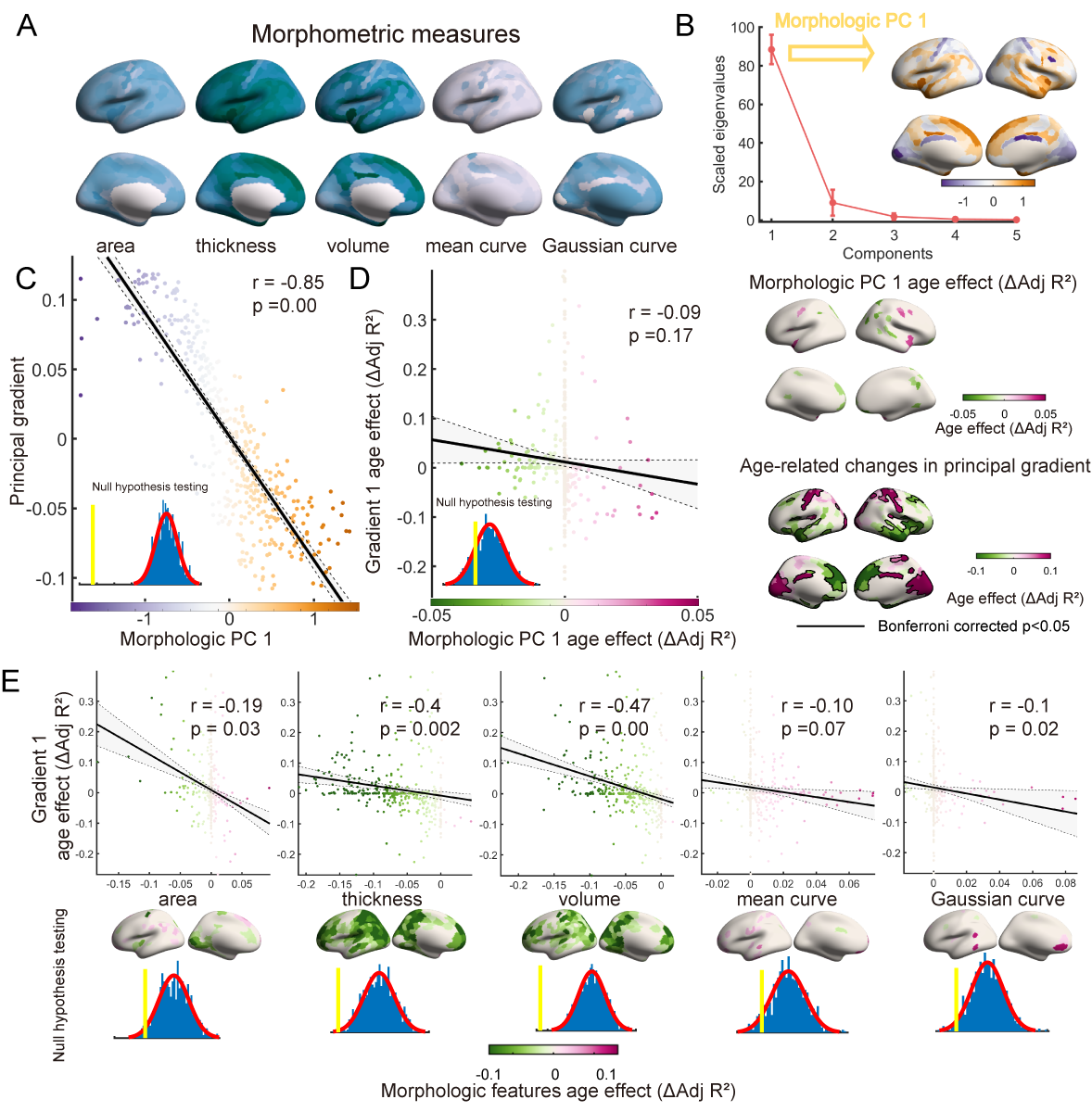

Supplement: S8 Fig — Results of the analysis of relationships between cortical morphological features using the independent HCP-D dataset for ages 6–14, employing the same steps as in the discovery dataset. (TIF) [file pbio.3002710.s008.tif]

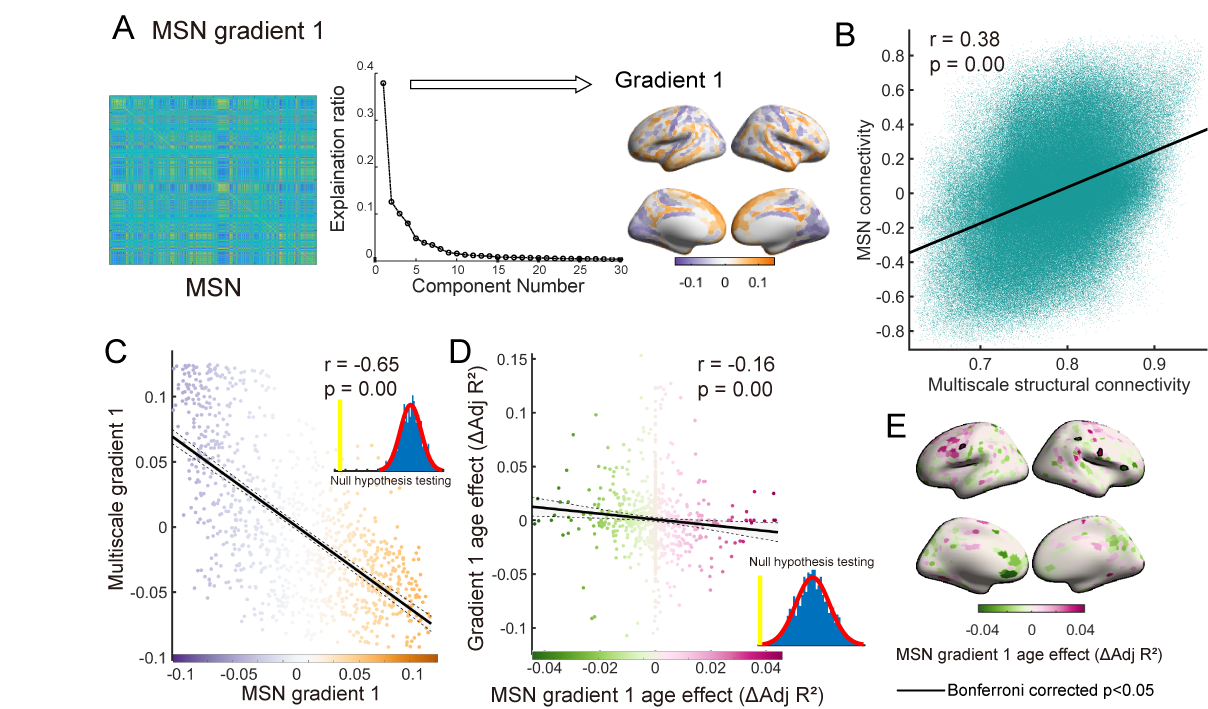

Supplement: S9 Fig — (A) The MSN was fed into diffusion map embedding algorithm. The first gradient captures the largest proportion of the variance. The group-averaged gradients were projected onto the cortical surface and visually represented (right). (B) The correlation between MSN edges and multiscale structural connectivity edges. (C) Spatial correlation between the multiscale structural principal gradient and MSN gradient 1. Each dot represents a brain node. The significance level was corrected for spatial autocorrelation (psurrogate = 0.00). (D) Spatial correlation between age-related ΔAdj R2-maps of the multiscale structural principal gradient and MSN gradient 1 (psurrogate = 0.00). (E) The spatial pattern of age-effect on the principal gradient, with the age effect quantified using ΔAdj R2. The results that survived Bonferroni correction are circled by black lines (Bonferroni corrected p < 0.05). (TIF) [file pbio.3002710.s009.tif]

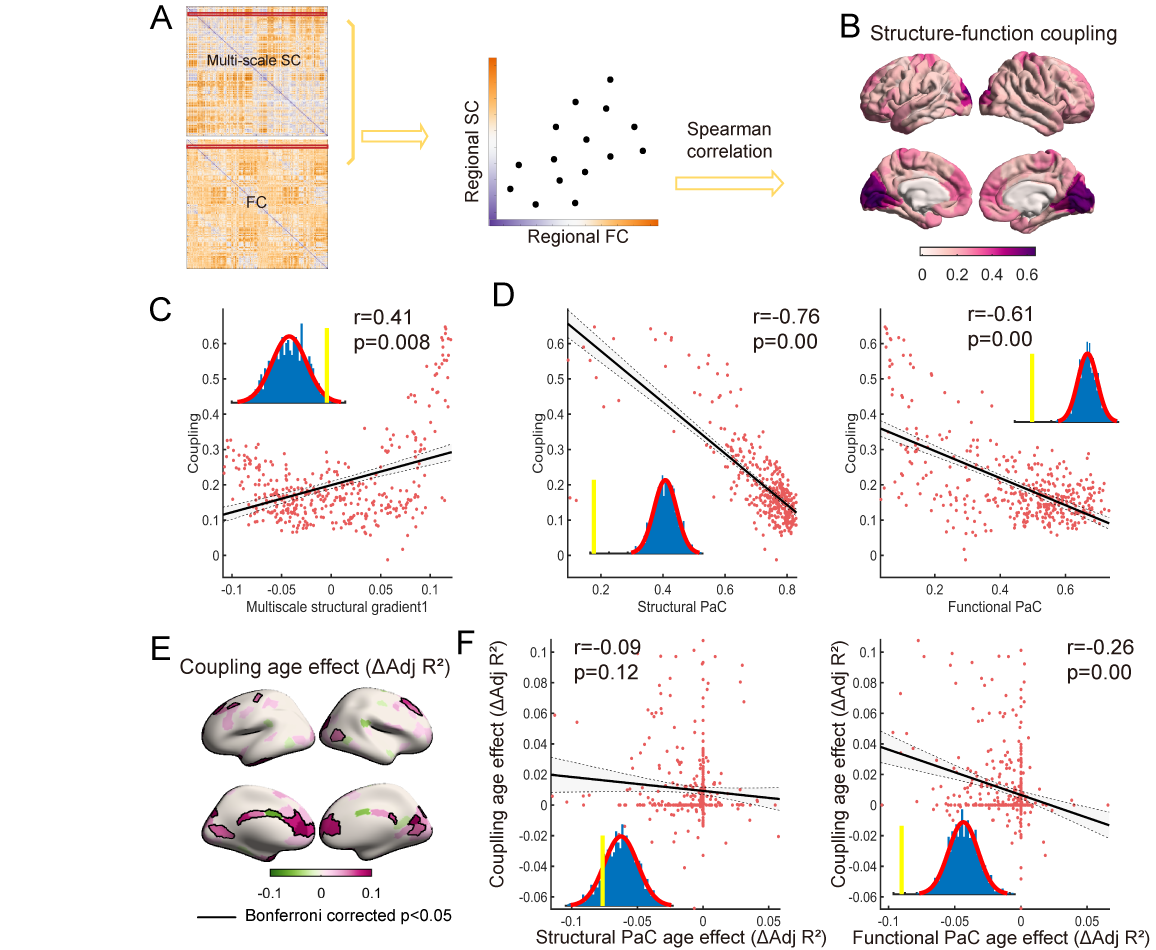

Supplement: S10 Fig — Results of the analysis of multiscale structure–function coupling using the independent HCP-D dataset for ages 6–14, employing the same steps as in the discovery dataset. (TIF) [file pbio.3002710.s010.tif]

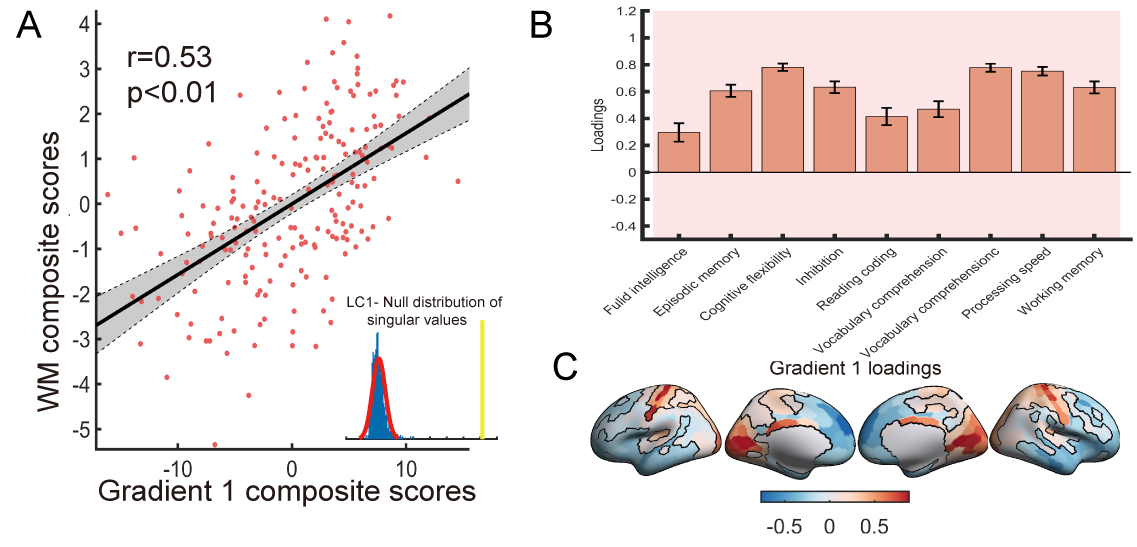

Supplement: S11 Fig — (A) Pearson correlations between the composite scores of the principal gradient and composite scores of cognitive measures. The inset figure shows the null distribution of singular values estimated by the permutation test (n = 1,000). (B) Loadings of cognitive measures were calculated by Pearson correlation between the cognitive measurements and their composite scores. The shadows represent significant loadings tested by bootstrap resampling (n = 1,000). Cognitive measures included fluid intelligence, episodic memory, executive function/cognitive flexibility, inhibition, language/reading decoding, vocabulary comprehension, processing speed, and working memory. (C) Gradient loadings were calculated by Pearson correlation between gradient 1 and their composite scores. The loadings of regions with black lines were subjected to a significance test by bootstrap resampling (n = 1,000). The data underlying this figure can be found at https://zenodo.org/records/14874537. (TIF) [file pbio.3002710.s011.tif]

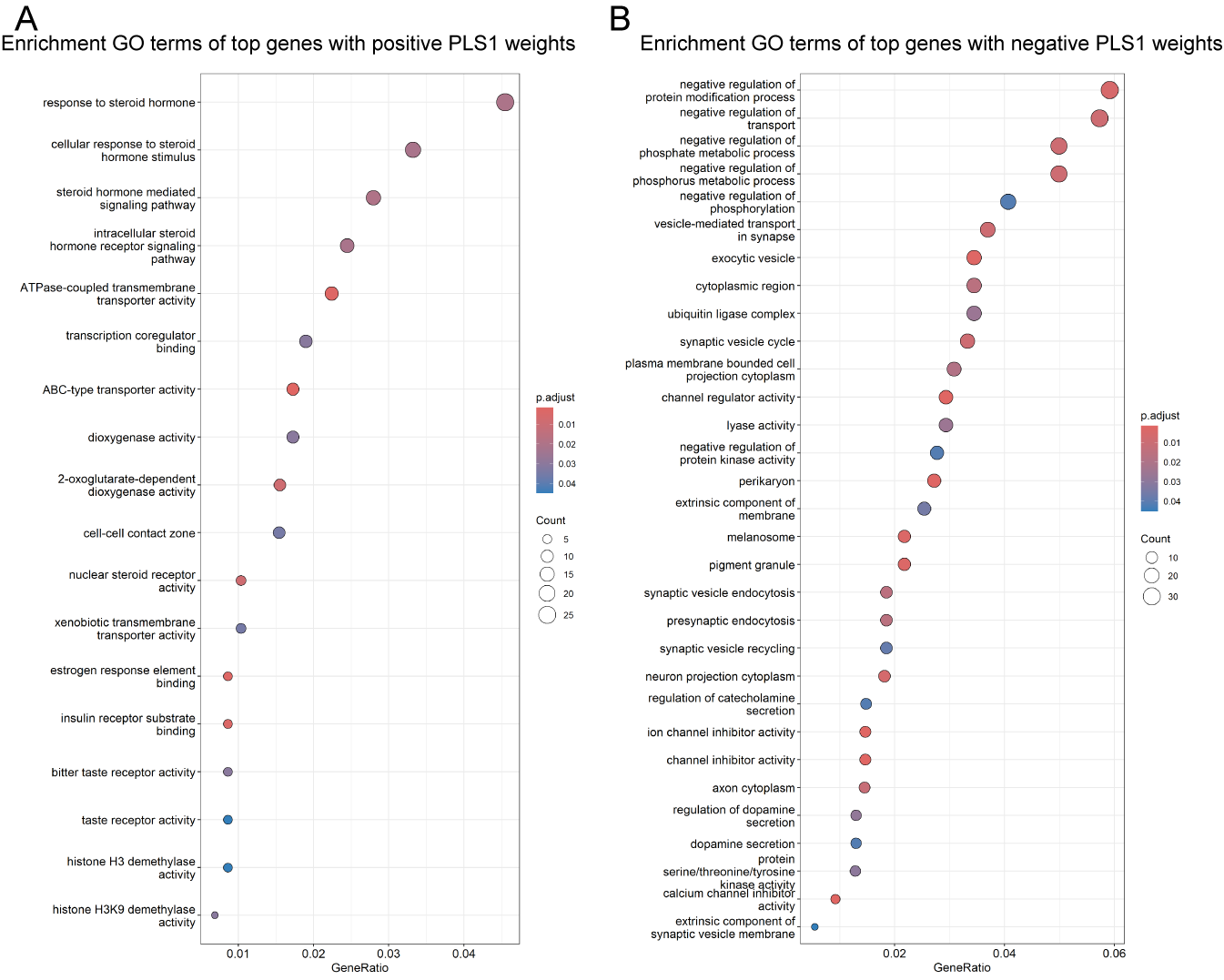

Supplement: S12 Fig — (A) Complete list of significant GO enrichment terms of top 10% genes with positive PLS 1 weights. (B) Complete list of significant GO enrichment terms of top 10% genes with negative PLS 1 weights (all p < 0.05, corrected for FDR and spatial autocorrelation). (TIF) [file pbio.3002710.s012.tif]

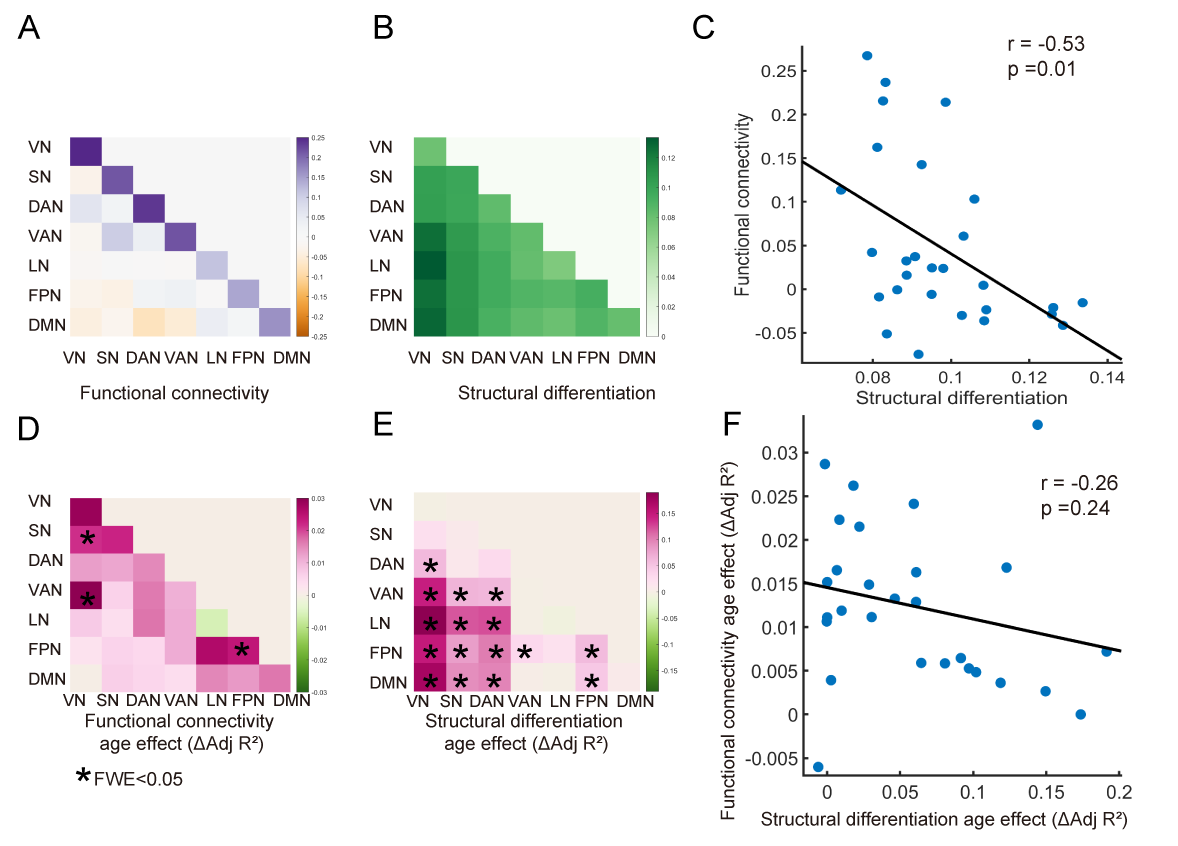

Supplement: S13 Fig — (A, B) The functional connectivity matrix/multiscale structural differentiation matrix was summarized according to Yeo functional networks [47]. (C) Correlation between multiscale structural differentiation and functional connectivity. (D, E) Age-effect on functional connectivity/multiscale structural differentiation, with the age-effect quantified using ΔAdj R2 and significant network reported with an asterisk (Bonferroni corrected p < 0.05). (F) The correlation between age effects on multiscale structural differentiation and age effects on functional connectivity. (TIF) [file pbio.3002710.s013.tif]

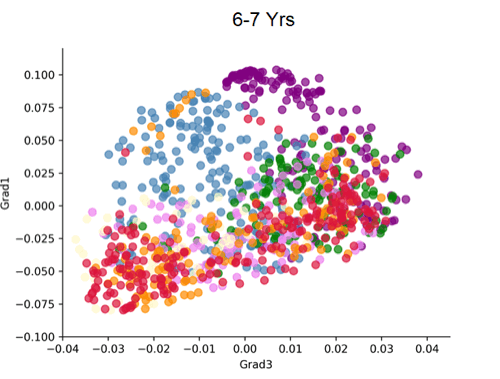

Supplement: S1 Movie — (GIF) [file pbio.3002710.s014.GIF]

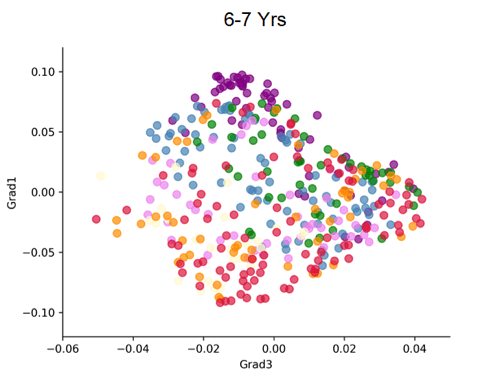

Supplement: S2 Movie — (GIF) [file pbio.3002710.s015.GIF]
