# Supplementary material for: The multiscale brain structural re-organization that occurs from childhood to adolescence correlates with cortical morphology maturation and functional specialization
Source: PLoS Biol. 2025 Apr 1;23(4):e3002710. doi: 10.1371/journal.pbio.3002710 (PMC12017512; doi:10.1371/journal.pbio.3002710)
Supplement: S1 Table — (DOCX) [file pbio.3002710.s016.docx]

|  | Original PLSC | | Cross-validated PLSC | | | |
| --- | --- | --- | --- | --- | --- | --- |
|  |  |  | Training data | | Testing data | |
|  | Corr | Permuted p | Corr(mean) | Permuted p(mean) | Corr(mean) | Permuted p(mean) |
| CBD WM LC1 | 0.46 | 0.001 | 0.51 | 0.005 | 0.22 | 0.04 |
| CBD ATT LC1 | 0.37 | 0.001 | 0.42 | 0.003 | 0.16 | 0.07 |
| HCP-D cognitive measures LC1 | 0.53 | 0.001 | 0.55 | 0.001 | 0.45 | 0.02 |

**S1 Table. The cross-validation results of Partial least square correlation (PLSC) analysis.** We conducted 10 iterations of 5-fold cross-validation, splitting our sample into training (80% of subjects) and testing (20% of subjects) sets for each iteration. The process for each fold involved performing PLSC analysis on the training set to obtain weights, which were then applied to the original data of the testing set for both brain (X) and behavioral (Y) measures to compute composite scores. We calculated the correlation (r-value) between the brain and behavior composite scores in the testing set. Statistical significance (permuted p-value) was determined by permuting the behavioral data 1000 times for each fold. The correlations and p-value were averaged across the folds and iterations.
