# Supplementary material for: The multiscale brain structural re-organization that occurs from childhood to adolescence correlates with cortical morphology maturation and functional specialization
Source: PLoS Biol. 2025 Apr 1;23(4):e3002710. doi: 10.1371/journal.pbio.3002710 (PMC12017512; doi:10.1371/journal.pbio.3002710)
Supplement: Attachment — Submitted filename: Response to Reviewers.docx [file pbio.3002710.s019.docx]

**Response to the editor:**
Having discussed the reviews with the Academic Editor, we think you should address all comments from the reviewers. Furthermore, we would also like you do discuss the limitations of dMRI tractography, including poor correlation with ground truth tract tracing, the abundance of false positives and the inability to measure the density of the inter-areal network.

**R**: We thank the editor for this critical point. Currently, dMRI tractography does face challenges in accurately reconstructing white matter connectivity, often resulting in false positives and negatives(1). Although streamline tractography aligns with fiber orientations, it does not correlate reconstructed connections with actual fiber density, making streamline counts non-quantitative measures of axonal numbers (2). In this study, we have employed advanced probabilistic fiber tractography utilizing single-shell 3-tissue constrained spherical deconvolution (SS3T-CSD) (3) and multi-shell multi-tissue spherical deconvolution (4) to derive the fiber orientation distribution. Furthermore, we applied anatomically constrained tractography (5) with the segmentation results of the structural MR images and spherical deconvolution-informed filtering of tracks (SIFT) approach (6) to enhance biological accuracy.

We have added the limitation of dMRI tractography into the discussion section as：
*“**Second, dMRI tractography faces challenges in accurately reconstructing white matter connectivity, often resulting in false positives and negatives (86). Although streamline tractography aligns with fiber orientations, it does not correlate reconstructed connections with actual fiber density, making streamline counts non-quantitative measures of axonal numbers (87).”* ***[Line 647-651]***

**Point-by-point response to Reviewer: 1**
This manuscript examines the development of structural connectivity gradients in a combined cross-sectional and longitudinal sample of children and adolescents using linear mixed effects models. Multiple analyses are performed. I can appreciate the utility of characterizing the maturation of structural connectivity along the SA axis.
However, the conceptual basis of the main analysis is problematic and the manuscript could benefit from more precise language.

Q1. The analysis rests on the combination of three different measures: structural connectivity (SC) measured with diffusion MRI, geodesic distances (GD) between pairs of regions, and microstructural profile covariance (MPC). The aggregation of these measures is proposed to constitute a model of cortical wiring. This is highly debatable. GD and MPC are not estimates of wiring. GD simply measures the distance between areas. Taking the inverse will emphasize short-range pairs, which are already over-emphasized in diffusion MRI. The use of MPC follows from evidence that areas with similar cytoarchitecture are more likely to be connected. This is a probabilistic statement and there is no guarantee that such areas will in fact be connected. SC, GD, and MPC are thus entirely different properties, with different ranges of values and different conceptual underpinnings. The rationale for their combination lacks a strong foundation, which makes it difficult to interpret highly abstract gradient embeddings of the concatenated matrices. The relative contributions of each to the results are also unclear. At a minimum, I suggest that the authors (a) provide a more compelling rationale for this model (other than citing prior work); and (b) try to parse the contributions of SC, GD, and MPC separately.

**R:** We thank the reviewer for the insightful comments. Your viewpoints have significantly enhanced our understanding of cortical wiring modeling. We have reevaluated our model's conceptual basis, focusing on the distinct roles of structural connectivity (SC), geodesic distances (GD), and microstructural profile covariance (MPC). To address the concern about the interpretation of gradient embeddings, we have conducted additional analyses to better delineate the relative contributions of SC, GD, and MPC. We hope to provide a clearer explanation of the rationale of our model as well as the distinct contributions of these individual features.

(a) In response to the reviewer’s concerns regarding the combination of structural connectivity (SC), geodesic distances (GD), and microstructural profile covariance (MPC) in this previously proposed “cortical wiring” model, we would like to further explain the rationality of this model.

(1) **Cortical wiring: Long-range and Short-range Neuronal Connections**

"Cortical wiring" refers to the connections between neurons, encompassing not only long-range pathways through the brain's white matter but also short-range connections within the gray matter (7). Cortical regions are interconnected through axons, projecting from one region to their target cortical region (8). Upon entering gray matter, each axon branches out to form one or more terminal arbors, synapsing with numerous postsynaptic neuronal processes in a specific laminar layer (8). Two distant brain regions can be linked by long fibers that depart from the cortex, known as deep white matter (DWM). Conversely, close-by regions, often neighboring gyri around the cortical sulci, are connected by short fibers referred to as superficial white matter (SWM) or U-fibers (8). These superficial white matter represent the majority of WM connections in the human brain (9), forming the last myelinated structures that mature no later than the fourth or fifth decade of life (10). A single axon can have a rather complex trajectory (11), potentially branching and terminating in multiple cortical regions (12, 13). Below is a schematic of our understanding of how cortical wiring is organized. The brain's white and gray matter collectively construct intricate neural connections, with the underlying structures determining the efficiency of information transmission and the realization of brain functions.

Figure 1. Schematic representation of cortical wiring organization. Upper left panel: neuronal connections within gray matter, showing neuronal cell bodies and axonal branches. Lower left panel: deep white matter schematic. Middle panel: coronal section schematic of the brain. Right panel: post-mortem stained brain slice image, with black lines representing superficial white matter (i.e., shallow white matter beneath the gray matter). Schematics adapted from (14-16).

(2) **The prevailing technique and its limitations of modeling cortical wiring**

*DMRI: Modeling deep white matter fibers.* Diffusion magnetic resonance imaging (dMRI) is a widely used technique for measuring the direction and integrity of fiber tracts within the brain. DMRI tractography is an advanced imaging technique that allows for in vivo reconstruction of the brain's white matter connections on a macro scale. Over the past two decades, the study of brain connectivity using dMRI tractography has played a prominent role in the neuroimaging research landscape. The application of tractography has facilitated the visualization of white matter tracts, primarily through a qualitative approach, which still holds an important position in today's clinical settings and neuroanatomical research (17).

*Modeling superficial white matter fibers:* However, diffusion MRI encounters challenges in accurately detecting superficial white matter fibers due to their intricate and complex nature, as well as their close proximity to gray matter, rendering their identification particularly difficult(18). Due to limitations in resolution and methods for handling fiber crossings, dMRI falls short in reconstructing superficial fibers effectively (19). Furthermore, most existing methods for identifying white matter tracts are tailored to large and stable deep white matter bundles, thus they do not transition well to the intricate nature of superficial white matter (18).

*Modeling connections within gray matter:* dMRI tracking terminates at the gray-white matter interface because of the relative lack of anisotropic water diffusion in the gray matter, preventing the measurement of axonal and dendritic connections within the gray matter (20).

(3) **Comprehensive structural connectivity features to enhance structural wiring modeling**

From the above, dMRI can be used to model the deep white matter fibers. However, modeling of superficial white matter and connections within gray matter are needed.

**GD** can measure cortical spatial proximity, which is particularly useful for predicting short-range cortico-cortical connections and can be used to simulate lateral connections within gray matter (14). It may reflect the wiring cost, enabling estimation of short-range connections within gray matter and superficial white matter. Based on neuroanatomical data on cat, a research showed that the majority of cortico-cortical connections can be well predicted by distances between the cortical regions (21).

**MPC**：Within the gray matter, myelin sheaths envelop some neuronal axons. In recent years, the T1w/T2w ratio has shown to be sensitive to myelin content, thus serving as a proxy for microstructure in gray matter (22). The recently proposed microstructure profile covariance (MPC) method could be used to model cortico-cortical connections based on the similarity of microstructure profiles sampled along cortical columns across brain regions, which has indicated its biological basis (23).

The “structural model” proposed in the 1990s posited that cortical regions with similar cytoarchitecture are more likely to be connected. In recent years, evidence from animal experiments and neuroimaging have validated this theory. For instance, a recent study using tracers and cellular staining techniques in cat, mouse, and macaque monkeys demonstrated a correlation between cytoarchitectural similarity and connection probability (24). In humans, research utilizing the ultrahigh-resolution BigBrain dataset and HCP dMRI data suggests that cortical regions with higher similarity in laminar cytoarchitectonic patterns have a greater likelihood of being connected (25). Therefore, the MPC approach may estimate structural connectivity probability by evaluating the similarity of microstructure profiles between gray matter regions, based on the "structural model" hypothesis that the likelihood of connections is closely related to the microstructural similarity of cortical areas (23, 26).

Therefore, GD and MPC features can enhance the modeling of cortical wiring. Recently, the multiscale structural wiring model was introduced by combining connectivity features across micro and macro scale, encompassing diffusion MRI tractography, GD, and MPC (27), which could comprehensively capture the complex connectivity characteristics of the brain.

**How to combine the features:** To balance the contribution of each input measure, the nonzero entries of the MPC, tract strength (TS) and inverted GD matrices were rank normalized and the less sparse matrices (GD and MPC) were rescaled to the same numerical range as the sparsest matrix (TS). This approach allows us to leverage the complementary information provided by TS, GD, and MPC, resulting in a model that better reflects the complexity of cortical wiring and offers improved insights for understanding complex brain connection patterns.

（4）**Neurobiological validation of the multiscale structural wiring model**

The neurobiological validity of the model has been demonstrated through a series of validation experiments (27). These experiments showed that the new wiring space reflects cortical microcircuit features, including pyramidal neuron depth and glial expression. Furthermore, the model enabled effective simulations of functional connectivity and dynamics based on resting-state functional magnetic resonance imaging (rs-fMRI) and human intracranial electroencephalography (EEG) coherence.

(b) We appreciate the reviewer's suggestion and have analyzed the individual contributions of the three features as recommended. Specifically, we calculated gradients based on the matrix of each individual feature and correlated them with the multiscale gradients, using the correlation coefficient as a measure of contribution. We selected the top three gradients at the group level for quantification. As shown in Figure 1, the x-axis represents the top three gradients of MPC, TS, and GD, while the y-axis represents the multiscale structural gradients. The top, middle, and bottom plots correspond to the top three multiscale structural gradients, respectively. The red dots represent the actual correlation coefficients between individual feature gradients and multiscale structural gradients. To correct for spatial autocorrelation, we generated 1000 surrogate maps that preserved the spatial autocorrelation of the given brain map using the variogram matching approach to estimate the significance. The box plots and density plots represent the null distribution of the 1000 null correlation coefficients. The results show that for the first multiscale structural gradient, all three features made significant contributions, with MPC contributing the most (r=0.93, p_surrogate_=0.00), followed by GD (r=-0.62, p_surrogate_=0.002), and TS (r=0.43, p_surrogate_=0.03) contributing the least. For the second gradient, only GD made a significant contribution (r=-0.95, p_surrogate_=0.00). For the third gradient, all three features also made significant contributions, with GD contributing the most (r=-0.73, p_surrogate_=0.00), while MPC (r=0.62, p_surrogate_=0.00) and TS (r=0.59, p_surrogate_=0.00) contributed to a less extent. The relatively lower contribution of TS might be due to the sparser nature of its matrix.

We have added these results to the paper as Supplementary S1 Fig, and revised the manuscript as follows:

*“To examine the contribution of each structural connectivity feature to the multiscale structural gradients, we employed the following steps. We calculated gradients based on the matrix of each individual structural connectivity feature, including TS, GD, and MPC, and correlated them with the multiscale gradients, using correlation coefficients as a measure of contribution. To focus our analysis, we selected the top three gradients at the group level for quantification. Recognizing the importance of accounting for spatial autocorrelation in brain maps, we generated 1000 surrogate maps that preserved the spatial autocorrelation of the given brain map using the variogram matching approach to estimate the significance (28) (**Supplementary* *S1 Fig). S1 Fig shows the correlations between the first three gradients of TS, GD, and MPC with the multiscale structural gradients, indicating that MPC and GD significantly contribute to the multiscale structural principal gradient, while TS contributes relatively less.”* ***[Line 818-828]***

**S1 Fig. Correlations between individual feature gradients (MPC, TS, GD) and multiscale structural gradients.** The x-axis represents the top three gradients of MPC, TS, and GD, while the y-axis represents the multiscale structural gradients. The top, middle, and bottom plots correspond to the top three multiscale structural gradients, respectively. Red dots indicate actual correlation coefficients. Spatial autocorrelation was corrected using 1000 surrogate maps generated via the variogram matching approach(28). Box plots and density plots show the null distribution of 1000 correlation coefficients.

Q2. The associations between SC maturation and cortical morphology follow logically from the observation that most aspects of brain development show a spatial pattern that resembles the SA axis (Sydnor et al. Neuron, 2021).
**R:** We appreciate the connection you've drawn between SC maturation, cortical morphology, and the spatial patterns along S-A axis observed in brain development as described by Sydnor et al. (2021). We agree that this perspective provides a logical framework for understanding our findings. We addressed this connection in the discussion of our manuscript as follows:

*“**Our findings revealed coordinated spatiotemporal developmental patterns of cortical morphometric profiles that encompass multiple morphometric features and the principal multiscale structural gradient incorporating white matter and cortical microstructure.* *This observation corresponds with the notion that most aspects of brain development, including the structural connectivity and cortical morphology, follow a spatial pattern resembling the S-A axis(29).* *In addition, from a more microscopic perspective, some empirical evidence and theoretical hypotheses have established associations between changes in cortical morphology and structural wiring;…”****[Line 547-554]***

Q3. The analysis presented in lines 323-349 showing that SC-FC coupling follows the SA-axis has already been demonstrated by several groups and the findings of the analysis of the participation coefficient follow from this fact.
**R:** We thank the reviewer for pointing this out, and apologize for the wording in our original manuscript. It's true that previous research has shown SC-FC coupling following the S-A axis. Our intention was to confirm these findings within the context of a more comprehensive multiscale structural connectivity model and to build upon them to further explore the implications for the participation coefficient. The coupling between this specific multiscale structural connectivity and FC has not been previously studied.

Regarding the participation coefficient analysis, we believe that it offers additional valuable information about the role of different brain regions in cortical organization. This analysis, in the context of the multiscale structural connectivity development, provides a more nuanced understanding of how structural and functional properties interact across the cortex during development.

To make the expression more appropriate, we have revised the ambiguous sentence in the original text as follows:

*“These findings align closely with the previous study, suggesting that the multiscale structure-function coupling reflected functional specialization and hierarchy (30).”****[Line 362-364]***

*“A previous study utilizing the white matter connectivity network and functional network demonstrated that their coupling reflects functional segregation* *(30). Consistent with this study, our study also revealed a significant spatial correlation between multiscale structure–function coupling and the PaC, as well as their interrelated developmental patterns. Our findings revealed that during development, regions exhibiting stronger coupling between multiscale structural and functional connectivity demonstrated stronger functional specialization, characterized by a greater degree of segregation.”* ***[Line 604-611]***

Q4. The PLS analysis should be cross-validated as within-sample effect size estimates are optimistic (Marek et al. Nature, 2022; Helmer, et al. Communications Biol, 2024).

**R:** We appreciate your insightful comments on the need for cross-validation, as highlighted by (31) and (32), to address the issue of optimistic within-sample effect size estimates. In the revised version, we have performed a cross-validation procedure to ensure the robustness of our results.

Specifically, we conducted 10 iterations of 5-fold cross-validation. For each iteration, we split our sample into training (80% of subjects) and testing (20% of subjects) sets. The process for each fold was as follows:

PLS analysis was first performed on the training set to obtain weights. These weights were then applied to the original data of the testing set for both brain (X) and behavioral (Y) measures to compute composite scores. We calculated the correlation (r-value) between the brain and behavior composite scores in the testing set. Statistical significance was computed by permuting the behavioral data 1000 times for each fold.

We also performed the same analysis steps on the Human Connectome Project - Development (HCP-D) dataset, with cognitive data including Fluid intelligence, Episodic memory, Executive Function/Cognitive Flexibility, Inhibition, Language/Reading Decoding, Vocabulary Comprehension, Processing Speed, and Working memory. We reached the same conclusion: the differentiation of the multiscale structural principal gradient is associated with better cognitive ability.

We have added the cross-validation results of CBD dataset to the main text and included the results from the HCP-D dataset in the supplementary materials. We have also added the steps for cross-validation to the methods section and emphasized the necessity of cross-validation. The revisions are as follows:

*“The findings from the replication dataset were largely consistent with the original results, encompassing various cognitive measures such as fluid intelligence, episodic memory, executive function/cognitive flexibility, inhibition, language/reading decoding, vocabulary comprehension, processing speed, and working memory (S11 Fig). Furthermore, we conducted 10 iterations of 5-fold cross-validation of the PLSC analysis (see Methods for further details). The mean correlation between cognitive and brain scores of test set was (WM: r=0.22, p=0.04; ATT: r=0.16, p=0.07 for discovery dataset; multiple cognitive measures: r=0.45, p=0.02 for replication dataset) (See S1 Table for more details). Collectively, these outcomes provide compelling evidence that the enhancement of the S-A axis pattern along multiscale structural gradient 1 is associated with improved cognitive performance.”****[Results section: Line 442-451]***

*“**Due to the issue of optimistic within-sample effect size estimates in PLSC analysis, as highlighted by Marek et al. (122) and Helmer et al. (123), we implemented a cross-validation procedure. We conducted 10 iterations of 5-fold cross-validation, splitting our sample into training (80% of subjects) and testing (20% of subjects) sets for each iteration. The process for each fold involved performing PLSC analysis on the training set to obtain weights, which were then applied to the original data of the testing set for both brain (X) and behavioral (Y) measures to compute composite scores. We calculated the correlation (r-value) between the brain and behavior composite scores in the testing set. Statistical significance was determined by permuting the behavioral data 1000 times for each fold.”****[Methods section: Line 984-992]***

**Fig 5.** **Partial least square correlation (PLSC) analysis revealed an association between the principal gradient and cognitive scores. (A, D)** Pearson correlations between the principal gradient and composite scores of working memory/attention. The inset figure shows the null distribution of singular values estimated by the permutation test (n = 1000). (**B, E)** Loadings of WM/attention were calculated by Pearson correlation between the cognitive measurements and their composite scores. The shadows represent significant loadings tested by bootstrap resampling (n=1000). **(C, F)** Gradient loadings were calculated by Pearson correlation between gradient 1 and their composite scores. The loadings of regions with black lines were subjected to a significance test by bootstrap resampling (n=1000).

**S11 Fig. Independent replication of PLSC analysis between the principal gradient and cognitive scores in HCD-D dataset. (A)** Pearson correlations between the composite scores of the principal gradient and composite scores of cognitive measures. The inset figure shows the null distribution of singular values estimated by the permutation test (n = 1000). **(B)** Loadings of cognitive measures were calculated by Pearson correlation between the cognitive measurements and their composite scores. The shadows represent significant loadings tested by bootstrap resampling (n=1000). Cognitive measures included fluid intelligence, episodic memory, executive function/cognitive flexibility, inhibition, language/reading decoding, vocabulary comprehension, processing speed, and working memory. **(C)** Gradient loadings were calculated by Pearson correlation between gradient 1 and their composite scores. The loadings of regions with black lines were subjected to a significance test by bootstrap resampling (n=1000).

We hope this comprehensive cross-validation strategy adequately addresses the reviewer's concern and strengthens the validity of our PLS analysis findings.

Q5. The gene enrichment analysis approach used here has been shown to be biased (Fulcher et al. Nature Comms, 2021). Appropriate null models should be used for inference.
**R:** Thank you for your concern regarding potential bias in our gene enrichment analysis approach. We acknowledge the important points raised by Fulcher et al. (33) about spatial autocorrelation as a key driver of false-positive bias in such analyses. To address this issue, we have implemented a null model approach as suggested.

Specifically, we first generated 1000 surrogate age-effected maps of the multiscale structural principal gradient using the variogram matching approach, preserving spatial autocorrelation (28). We then applied partial least squares (PLS) regression to identify the most relevant genes for each surrogate map, resulting in 1000 surrogate ordered gene sets. Subsequently, we performed Gene Ontology (GO) enrichment analysis on each surrogate gene set to obtain a null distribution of surrogate P-values for each GO term. Finally, we compared the empirical GO term p-value to this null distribution, considering it significant only if it was smaller than 95% of the surrogate P-values. This rigorous approach allowed us to robustly identify age-related genetic associations while accounting for spatial autocorrelation and controlling for false positives. GO enrichment analysis was performed using the clusterProfiler package (<https://bioconductor.org/packages/release/bioc/html/clusterProfiler.html>).

We have modified this section in our results and added corresponding steps to the methods section, emphasizing the necessity of using null models to correct for spatial autocorrelation. The modifications are as follows:

*“The significance of the GO enrichment terms was further evaluated using a null model, created by conducting enrichment analysis based on surrogate maps that preserved the empirically observed spatial autocorrelations (55). As shown in Fig 6D, several meaningful puberty hormones-related terms emerged for the PLS1+ genes, such as “steroid hormone-mediated signaling pathway”, “response to steroid hormone”, “intracellular steroid hormone receptor signaling pathway” and “cellular response to steroid hormone stimulus” (Fig 6D). On the other hand, the PLS1- genes were enriched in several synapse-related terms, such as “vesicle-mediated transport in synapse”, “synaptic vesicle endocytosis”, “axon cytoplasm”, and inhibition related terms, such as “channel inhibitor activity”, and “calcium channel inhibitor activity” (Fig 6E) (all p<0.05, corrected for FDR and spatial autocorrelation). For a complete list of significant GO terms, refer to Supplementary S12 Fig.”* ***[Results section: Line 485-496]***

*“**The genes with positive and negative PLS1 weights were then separately entered into gene enrichment analysis using clusterProfiler package (54). GO analysis was used to search for specific molecular function, biological process, and cellular component terms. Next, we adopted a null model approach, recognizing that spatial autocorrelation significantly drives false-positive bias in gene enrichment analysis. Specifically, we created 1000 surrogate age-effected maps of the multiscale structural principal gradient using the variogram matching method to maintain spatial autocorrelation (55). We then utilized PLS regression to identify the most relevant genes for each surrogate map, generating 1,000 null gene sets. Following this, we carried out GO enrichment analysis on each surrogate gene set, producing a null distribution of surrogate p-values for each GO term. Finally, we assessed the empirical GO term p-values against this null distribution, considering a result significant only if it fell below 95% of the surrogate p-values.”****[Methods section: Line 948-959]***

**Fig 6**. Association between age-related changes in the principal gradient and gene expression profiles. (A) Gene expression profiles across 416 brain regions. (B) The explained ratios for the first 10 components derived from the partial least squares regression analysis between age-related changes in the principal gradient and gene expression profiles. The first component (PLS1) accounted for the largest proportion of the variance and is depicted in the right panel. (C) Spatial correlation between age-related changes in the multiscale structural principal gradient and PLS1 scores. Each dot represents a brain region. The significance level was corrected for spatial autocorrelation (p _surrogate_=0.01). (D, E) Gene Ontology (GO) enrichment terms of the top 10% of genes with positive/negative PLS1 weights.

**S12 Fig.** **Association between age-related changes in principal gradient and gene expression profiles. (A)** Complete list of significant GO enrichment terms of top 10% genes with positive PLS 1 weights. **(B)** Complete list of significant GO enrichment terms of top 10% genes with negative PLS 1 weights (all p<0.05, corrected for FDR and spatial autocorrelation).

Q6. The manuscript would benefit from greater precision and clarity of language. For example:

Line 32- "reorganization of large-scale anatomical wiring" could mean anything. Please be specific

**R:** We have revised this sentence as:
*“From childhood to adolescence, the structural organization of the* *human brain undergoes dynamic and regionally heterogeneous changes across multiple scales, from synapses to macroscale white matter pathways.”* ***[Line 30-32]***

Line 37 - the use of GD, MPC, etc means that the model is not one of cortical "wiring"

**R:** We have revised this sentence as:
*“Here, using two independent multimodal imaging developmental datasets aged 6 to 14 years, we investigated developmental process of multiscale cortical organization by constructing an in vivo multiscale structural connectome model incorporating white matter tractography, cortico–cortical proximity, and microstructural similarity.”* ***[Line 34-38]***

Line 45 - why is multiscale used so often. Only one scale is considered - that defined by the parcellation.
**R:** Thank you for your insightful comment regarding the use of the term "multiscale" in our manuscript. We understand the concern about its usage, particularly in reference to the parcellation. As described in Q1, "cortical wiring" refers to the intricate connections between neurons in the brain, encompassing both long-range pathways through white matter and short-range connections within gray matter. To model cortical wiring, the initial multiscale structural wiring model incorporates dMRI tractography for estimating the macroscale white matter tracts, cortical geodesic distance (GD) for predicting short-range connections within gray matter and superficial white matter, and microstructural profile covariance (MPC) for estimating structural connectivity probability based on cytoarchitectural similarity in gray matter regions. Our intention with "multiscale" is to highlight that our structural connectivity features, including tract strength (TS), GD, and MPC, span different scales—ranging from microstructural to macrostructural aspects, rather than referring to multiple resolutions in parcellation.

Line 71 - it is unclear how the brain can "exhibit" "principles"

**R:** We have revised this sentence as:
*“The human brain is a complex network that follows coordinated structural organizational principles at multiple spatial scales.”* ***[Line 71-72]***

Line 75 - It is unclear what it means for "multiscale organization" to be "embedded within complex biological mechanisms"

**R:** We have revised this sentence as:

*“**Multiscale structural organization emerges from complex biological mechanisms and serves as a foundational framework to support various brain functions.”* ***[Line 74-76]***

Line 88 - GD does not quantify wiring cost. It is sometimes used as an estimate, but carries many assumptions with this usage.

**R:** We have revised this sentence as:
*“**The GD captures spatial proximity of the cortex which may estimate wiring cost.”* ***[Line 88]***

Line 89 - MPC does not evaluate the strength of structural connections. It measures MPC, which may or may not be related to the probability of connectivity between two areas.
**R:** Thank you for your insightful comment. We acknowledge that MPC does not directly measure the strength of structural connections. To address this, we have revised the text for clarity:

*“**Moreover, microstructural similarity is linked to connection probability in non-human animals, and the cortico-cortical “structural model” posits a close association between connectivity likelihood and similarity of microstructure across cortical regions (9, 10). The MPC assesses this similarity between cortical regions by systematically comparing the myelin-sensitive neuroimaging profiles in cortical columns (11).”* ***[Line 90-94]***

Line 92 - Please specific how or why these features "enhance" connectivity.

**R:** We have revised this paragraph as:

*“Diffusion MRI (dMRI) tractography is the predominant method for inferring white matter tracts, particularly deeper white matter tracts, but currently has limitations in detecting short-range connections within gray matter and superficial white matter fibers (6, 7). The GD captures spatial proximity of the cortex which may estimate wiring cost, enabling estimation of short-range connections in gray matter (8) and superficial white matter. Moreover, microstructural similarity is linked to connection probability in non-human animals, and the cortico-cortical “structural model” posits a close association between connectivity likelihood and similarity of microstructure across cortical regions (9, 10). The MPC assesses this similarity between cortical regions by systematically comparing the myelin-sensitive neuroimaging profiles in cortical columns (11). By incorporating GD and microstructural similarity as additional structural connectivity features, this multiscale structural model compensates for the limitations of relying solely on dMRI tractography (5). ”****[Line 85-96]***

Line 98 - unclear usage of "feature transition"
**R:** We have revised this sentence as:

*“**This axis represents continuous transitions of cortical properties across the cortical mantle from primary to association regions, capturing a hierarchical organization that manifests in anatomy (34), function(35), and evolution(36).”* ***[Line 101-103]***

Lines 173-174 - unclear what "progressively distributed toward both ends during development" means
**R:** We have revised this sentence as:

*“**Our results demonstrated that from the 6-7 age group to the 12-13 age group, the range of the principal gradient expands bilaterally (Fig 1B).”* ***[Line 176-177]***

Line 177 and several other places in the manuscript refer to increases/decreases of the principal gradient. I believe the authors are referring to increases/decreases in the range of values. The analysis are very abstract and the authors should be clear about what they mean; e.g., an expansion of the poles of the gradient? Increases for regions/networks that are not at the poles are relative to the poles; e.g., an increase in DAN values on its own is not interpretable. Its relative distance from one pole of the other is what should be interpreted.
**R:** We appreciate the insightful feedback regarding the interpretation of our results. We acknowledge the need for clarity regarding the terminology used to describe changes in the principal gradient. Actually, the reported increases and decreases refer to isolated changes in specific regions or networks, and these changes of these gradient values, especially the poles regions of the gradient, together cause the change of the gradient range. Moreover, we acknowledge the importance of emphasizing that changes in regions or networks not located at the poles should be understood relative to the poles themselves. To enhance conciseness and clarity, we have implemented the following modifications to the text:

*“**The network analysis also demonstrated an age-related expansion of the principal gradient, manifested by increased gradient scores in regions with high gradient scores (SN, VN) and the dorsal attention network (DAN), while scores decreased in regions with low gradient scores (ventral attention network (VAN), limbic network (LN), frontoparietal network (FPN), and DMN). These findings were corroborated by results from the laminar differentiation atlas (Fig 1C, right panel).”* ***[Line 179-184]***

Line 208 - it is unclear precisely what an explanation ratio quantifies.

**R:** We have revised this sentence as:
*“**The explanation ratio quantified the percentage of connectivity variance accounted for by that gradient, and a higher value signified a more prominent role in the organization of the structural connectome.”* ***[Line 212-214]***

Lines 209 - 210 - extremes and consistency of what?

**R:** We have revised this sentence as:
*“**The range indicated differentiation between the regions at the gradient extremes, and the standard deviation measured the heterogeneity of the gradient scores across regions.”* ***[Line 214-216]***

Line 218 - the dominance of the principal gradient would be revealed by the variance explained.
**R:** We have revised this sentence as:
*“These findings indicated a shift toward a more distributed structural network topology during development, with the principal gradient increasingly differentiating between primary and transmodal regions. This finding was consistent with the increasing dominance of the principal gradient.”****[Line 225-228]***

**Point-by-point response to Reviewer: 2**
The continuous differentiation of multiscale structural gradients from childhood to adolescence correlates with the maturation of cortical morphology and functional specialization

The authors built multimodal connectomes based on SC, geodesic distance mapping and microstructural similarity and then derived gradients of these. Age related changes were assessed in a longitudinal dataset aged from 6-14 years. They observed that the gradients spanned SA and AP axes, and they observed age related expansion of gradient space. Findings were shown to coincide with changes in cortical morphology and the authors observed associations to functional and cognitive measures.

This is overall an interesting study, taking advantage of longitudinal data, an advanced model of cortical wiring, and integrative methods that bridge different connectivity metrics, structural MRI, as well as functional and behavioral markers. The work demonstrates a noteworthy expansion of multiscale gradient pattern in the transition from childhood to adolescence, in particular along the sensory-association axis, and thus represents a logical extension of prior studies assessing multiscale gradient reconfigurations in later developmental stages.

I only have a few comments and suggestions:
Q1. In the reporting of the results, please provide measures of effect sizes and t-values together with the p-values, for both significant and non-significant effects. Please consistently indicate which findings were corrected for multiple comparisons and which ones weren't.
**R:** Thank you for your valuable feedback. We have addressed your concerns as follows:

In the revised version, we have included effect sizes alongside p-values for both significant and non-significant effects throughout the results section. Furthermore, we have consistently indicated which findings were corrected for multiple comparisons and which were not, specifying the correction method used (e.g., FDR, Bonferroni) or explicitly stating when results are uncorrected.

These changes have been implemented throughout the manuscript to enhance the clarity and interpretability of our results.

Q2. The expansion of the multiscale pattern in childhood is very interesting. Coyuld the authors perform supporting analyses to determine which of the constituent features drives the overall findings the most? I assume geodesic distance relationships to change least while MPC and SC are likely changing more in that age range.
**R:** We appreciate the reviewer’s suggestion to investigate which constituent features drive the overall findings most significantly. To address this, we have conducted additional analyses as follows:

We calculated the first three gradients for each structural connectivity matrix based on individual feature. Then we applied the same GAMM model to characterize the developmental effects at the brain region level for each single-feature gradient. We then correlated the brain maps of developmental effects obtained from single-feature gradients with the developmental effect brain map of the multiscale structural gradients. We used the resulting correlation coefficients to quantify the contribution of each single feature to the overall gradient.

As shown in S7 Fig, the x-axis represents the developmental effects of the first three gradients of MPC, TS, and GD, while the y-axis represents the developmental effects of the multiscale structural gradients. The top, middle, and bottom plots correspond to the top three multiscale structural gradients, respectively. The red dots indicate the actual correlation coefficients between the developmental effects of single-feature gradients and the developmental effects of multiscale structural gradients. To correct for spatial autocorrelation, we generated 1000 surrogate maps that preserved the spatial autocorrelation of the given brain map using the variogram matching approach to estimate the significance. The box plots and density plots represent the null distribution of the 1000 null correlation coefficients. Our results indicate that for the first multiscale structural gradient, all three features made significant contributions, with MPC contributing the most (r=0.98, p_surrogate_=0.00), followed by GD (r=-0.59, p_surrogate_=0.001), and TS (r=-0.25, p_surrogate_=0.02) contributing the least. For the second gradient, GD made a significant contribution (r=-0.51, p_surrogate_=0.00), followed by MPC (r=0.20, p_surrogate_=0.00). For the third gradient, only MPC made significant contribution (r=0.86, p_surrogate_=0.00). Thus, during development of 6-14 years old, the differentiation along the multiscale structural principal gradient is driven by the MPC most, followed by GD, and TS drives least. The relatively lower contribution of TS might be due to the sparser nature of its matrix.

We have included these new analyses and results in the S7 Fig. We hope this addition satisfactorily addresses your query.

We added this analysis as:

*“To examine which structural connectivity features drive the development of the multiscale structural gradients, we performed same analysis based on the age-effect maps of the first three multiscale structural gradients and individual feature gradients. As shown in S7 Fig, the development of MPC and GD significantly contributes to the development of the multiscale structural principal gradient, while TS contributes relatively less to the multiscale structural principal gradient.”* ***[Line 829-834]***

**S7 Fig.** Correlations between developmental effects of individual feature gradients (MPC, TS, GD) and multiscale structural gradients. The x-axis represents the developmental effects of top three gradients of MPC, TS, and GD, while the y-axis represents the developmental effects of multiscale structural gradients. The top, middle, and bottom plots correspond to the top three multiscale structural gradients, respectively. Red dots indicate actual correlation coefficients. Spatial autocorrelation was corrected using 1000 surrogate maps generated via the variogram matching approach. Box plots and density plots show the null distribution of 1000 correlation coefficients.

We believe this additional analysis provides valuable insights into the driving forces behind the multiscale pattern expansion and addresses your question regarding the relative contributions of GD, MPC, and TS.

Q3. Figure 3E - I assume findings are corrected for spatial autocorrelation? Please comment and/or adjust as you did for eg Figure 3C. Ditto for findings in F4 D and F4F. Would also co-plot the null distributions for these plots just to make presentation consistent.
**R:** We thank the reviewer for pointing out this issue. We have addressed your concerns as follows:

Figure 3E: We've clarified that these findings are corrected for spatial autocorrelation.

Figure 4D and 4F: We've applied the same spatial autocorrelation correction and indicated this in the captions and text.

We've co-plotted null distributions for Figures 3E, 4D, and 4F to maintain consistency with Figure 3C.

We hope these changes improve the clarity and consistency of our results presentation.

Q4. What is the rationale to specifically assess WM and attentional ability. I understand these are domains that change but are the findings specific to those domains? A broader assessment of cognitive measures (if available in the dataset) could be worthwhile here to show which domains relate and which dont relate.

**R:** We thank the reviewer for this insightful comment. Due to the limited cognitive measures, our initial analysis focuses solely on two cognitive domains, i.e. working memory and attention ability. To address reviewer’s concern and provide a more comprehensive assessment, we conducted an additional analysis using the Human Connectome Project - Development (HCP-D) dataset. This dataset offers a broader range of cognitive measures, including Fluid intelligence, Episodic memory, Executive Function/Cognitive Flexibility, Inhibition, Language/Reading Decoding, Vocabulary Comprehension, Processing Speed, and Working memory. Consistent with the CBD dataset, we explored the relationship between cognitive scores and the multiscale structural principal gradient across individuals using the HCP-D dataset via Partial Least Squares Correlation (PLSC) analysis. Through PLSC, we generated latent components (LCs) that captured the optimal associations between the principal gradient and cognitive scores. The first LC (LC1) exhibited significance in the permutation test (p<0.01). The correlation between the cognitive composite score and the gradient 1 composite score was significant, indicating a strong positive relationship between the cognitive and gradient data (r=0.53, p<0.01) (S11 Fig A). As shown in S11 Fig B, all of the cognitive scores are significant. Combining S11 Fig C, better cognitive scores were associated with higher gradient 1 values in primary regions and lower values in transmodal regions.

Thus, our analysis of the HCP-D dataset yielded the same conclusion: the differentiation of the multiscale structural principal gradient is associated with better cognitive ability across multiple domains. These results, which support and extend our original findings, have been included in the supplementary materials for a more comprehensive view of the relationship between brain structure and cognitive function.

This additional analysis not only addresses the limitations of our initial dataset but also demonstrates the robustness of our findings across a wider range of cognitive domains. We hope this provides stronger evidence for our findings and addresses your concern about the specificity of our initial findings to working memory and attentional ability.

**S11 Fig. Independent replication of PLSC analysis between the principal gradient and cognitive scores in HCD-D dataset.** (A) Pearson correlations between the composite scores of the principal gradient and composite scores of cognitive measures. The inset figure shows the null distribution of singular values estimated by the permutation test (n = 1000). (B) Loadings of cognitive measures were calculated by Pearson correlation between the cognitive measurements and their composite scores. The shadows represent significant loadings tested by bootstrap resampling (n=1000). Cognitive measures included fluid intelligence, episodic memory, executive function/cognitive flexibility, inhibition, language/reading decoding, vocabulary comprehension, processing speed, and working memory (C) Gradient loadings were calculated by Pearson correlation between gradient 1 and their composite scores. The loadings of regions with black lines were subjected to a significance test by bootstrap resampling (n=1000).

Q5. I wonder whether the gene expression correlations adds much to the paper, given that AHBA is mainly based on adults and that the sample size is small. These findings are of course not redundant, but the authors could consider moving those somewhat exploratory analyses to the supplements.
**R:** We understand the reviewer's concerns about the limitations of the AHBA dataset. We acknowledge that the AHBA dataset is primarily based on adult samples and has a small sample size, and we have discussed the limitation in the discussion section. However, the gene expression correlation analyses provide valuable insights into the biological underpinnings of our findings. Specifically, after correcting for spatial autocorrelation using a spatial null model, our analyses revealed stable associations between the development of the multiscale structural principal gradient and hormone, synapse, axon, and inhibition-related GO terms. These findings help bridge the gap between observed neuroimaging changes and underlying biological processes, providing potential mechanistic explanations for the developmental patterns we observed. Therefore, we believe these analyses, despite their limitations, contribute meaningful biological context to our main findings and would prefer to keep them in the main text.

Q6. For independent verification, the anonymized raw and processed should ideally be made openly accessible, as longitudinal data in that age range is currently lacking. Alternatively, or in addition, the findings could be strengthened if results were replicated eg using the open access ABCD dataset (which has longitudinal data starting at 9 yo).
**R:** We appreciate the reviewer's suggestion regarding independent verification of our results. While our dataset will be made publicly available in the future, we understand the importance of addressing this concern promptly. Given the age range discrepancy between our dataset (6-14 years) and the ABCD Study (which begins at ages 9-10), we leveraged the Human Connectome Project Development (HCP-D) dataset as an independent validation cohort, as it encompasses the age range of our study population (6-14 years) and allows for comprehensive validation of our findings. The HCP-D dataset provides multi-modal neuroimaging data, including T1w, T2w, dMRI, and rs-fMRI, allowing for a comprehensive validation of our results across multiple imaging modalities. We conducted analyses on the HCP-D dataset that parallel our original study. We first characterized the developmental patterns of multiscale structural gradients during childhood and adolescence at whole brain level and regional level (S5 Fig). Furthermore, we explored the associations between the principal gradient and the cortical morphology as well as their development interaction patterns (S8 Fig). We also examined the associations between development of multiscale structure–function coupling and the change of participation coefficient (S10 Fig). The results obtained from this independent dataset are consistent with our original findings, thus providing additional support for the robustness of our conclusions.

We believe this approach addresses the reviewer's concern for independent verification while also strengthening the overall reliability of our results. The details of these validation analyses using the HCP-D dataset have been added to the supplementary materials of our manuscript.

**S5 Fig. Independent replication of multiscale structural gradients during childhood and adolescence based on Schaefer 400 atlas.** The same steps were conducted to regenerate the multiscale structural gradients using an independent HCP-D dataset aged 6-14 years old.

**S8 Fig. Independent replication of association between the multiscale structural principal gradient and morphometric features.** Results of the analysis of relationships between cortical morphological features using the independent HCP-D dataset for ages 6-14, employing the same steps as in the discovery dataset.

**S10 Fig. Independent replication of multiscale structure–function coupling during development.** Results of the analysis of multiscale structure–function coupling using the independent HCP-D dataset for ages 6-14, employing the same steps as in the discovery dataset.

**Point-by-point response to Reviewer: 3**
This paper used multiscale structural architecture gradients to investigate the developmental process from childhood to adolescence (aged 6 to 14 years). The authors used an in vivo model of cortical wiring that combines features of white matter tractography, geodesic distance, and microstructural similarity to construct a multiscale brain structural connectome. Furthermore, the authors studied the relationships between the development process of multi-scale structural gradient space and cortical morphometric features, functional interactions, cognitive behavior, and gene expression profiles.
Overall, I would fully endorse the publication if the following major concerns can be addressed:

Q1. A recent study by Casey et al. (2020) proposed an in vivo structural wiring model to characterize comprehensive neural organizations across multiple scales. This model integrates various neuroimaging features to provide a detailed understanding of brain structure connectivity. Specifically, the authors focused on three key features: tract strength (TS) at the macroscale, cortical geodesic distance (GD), and microstructural profile covariance (MPC). Indeed, regarding the integration of additional features such as Morphometric Similarity Network (MSN) or Morphometric INverse Divergence (MIND), these methods introduced by Seidlitz et al. (2018) and Sebenius et al. (2023), respectively. Why did the authors use only three neuroimaging features to build an in vivo multi-scale structural wiring? What are the results of the development process of multi-scale structural gradients combining four neuroimaging features (i.e., TS, GD, MPC, MSN (or MIND))? Including MSN or MIND in analyses would likely enhance the model's ability, thereby enriching the brain's characterization of multiscale structural wiring.

**R:** We thank the reviewer for this thoughtful comment on the choice of neuroimaging features for the multiscale structural wiring model. We appreciate the suggestion to consider additional features like MSN or MIND.

MSNs are constructed by representing each brain region as a vector that includes multiple MRI-derived features, such as cortical thickness, surface area, volume, and curvature(37). The similarity between regions is calculated by assessing the pairwise correlation between these feature vectors. Research has consistently demonstrated that MSNs exhibit high levels of robustness, reproducibility, and reliability (38). Growing evidence suggests that genetic, cytoarchitectonic, and chemoarchitectonic factors play a significant role in the formation and shaping of MSNs (38). Following your suggestion, we have now included MSN as a fourth feature and recalculated the multiscale gradient using TS, GD, MPC, and MSN. We have characterized the developmental pattern based on this four-feature model. We demonstrated the spatial distribution patterns of group-level gradients. As shown in the below figure, the first two gradients are consistent with the previous model's patterns. The first gradient reflects the transition from primary to transmodal regions, and the second gradient differentiates the left and right hemispheres (refer to Question 2 for the previous model's gradient patterns). However, the third gradient differentiates primary regions and transmodal regions, similar to the first gradient. This differs from the previous model's third gradient, which differentiated between anterior and posterior cortices. Using GAMMs, we characterized the development of gradients at the brain region level. We found that the development of the first gradient is consistent with previous models, showing differentiation along the principal gradient. The development of the second gradient is also similar to the previous model, showing a weakening differentiation between the left and right hemispheres. The third gradient shows an increase in the visual area.

Figure 2. (A) The first three group-level gradients derived from the structural connectivity model that includes TS, GD, MPC, and MSN. (B) The spatial pattern of age-effect on the first three gradients, with the age effect quantified using ΔAdj R2. The results that survived Bonferroni correction were circled by black lines (Bonferroni corrected p<0.05).

These additional analyses offered interesting insights. However, we chose to focus on three neuroimaging features — tract strength (TS), cortical geodesic distance (GD), and microstructural profile covariance (MPC) — based on their biological basis on cortical wiring. Below are our considerations in multiscale structural wiring modeling:

**(1) Cortical wiring: Long-range and Short-range Neuronal Connections**

"Cortical wiring" refers to the connections between neurons, encompassing not only long-range pathways through the brain's white matter but also short-range connections within the gray matter (7). Cortical regions are interconnected through axons, projecting from one region to their target cortical region (8). Upon entering gray matter, each axon branches out to form one or more terminal arbors, synapsing with numerous postsynaptic neuronal processes in a specific laminar layer (8). Two distant brain regions can be linked by long fibers that depart from the cortex, known as deep white matter (DWM). Conversely, close-by regions, often neighboring gyri around the cortical sulci, are connected by short fibers referred to as superficial white matter (SWM) or U-fibers (8). These superficial white matter represent the majority of WM connections in the human brain (9), forming the last myelinated structures that mature no later than the fourth or fifth decade of life (10). A single axon can have a rather complex trajectory (11), potentially branching and terminating in multiple cortical regions (12, 13).

**(2) The prevailing technique and its limitations of modeling cortical wiring**

*DMRI: Modeling deep white matter fibers.* Diffusion magnetic resonance imaging (dMRI) is a widely used technique for measuring the direction and integrity of fiber tracts within the brain. DMRI tractography is an advanced imaging technique that allows for in vivo reconstruction of the brain's white matter connections on a macro scale. Over the past two decades, the study of brain connectivity using dMRI tractography has played a prominent role in the neuroimaging research landscape. The application of tractography has facilitated the visualization of white matter tracts, primarily through a qualitative approach, which still holds an important position in today's clinical settings and neuroanatomical research (17).

*Modeling superficial white matter fibers:* However, diffusion MRI encounters challenges in accurately detecting superficial white matter fibers due to their intricate and complex nature, as well as their close proximity to gray matter, rendering their identification particularly difficult (18). Due to limitations in resolution and methods for handling fiber crossings, dMRI falls short in reconstructing superficial fibers effectively (19). Furthermore, most existing methods for identifying white matter tracts are tailored to large and stable deep white matter bundles, thus they do not transition well to the intricate nature of superficial white matter (18).

*Modeling connections within gray matter:* dMRI tracking terminates at the gray-white matter interface because of the relative lack of anisotropic water diffusion in the gray matter, preventing the measurement of axonal and dendritic connections within the gray matter (20).

(3) **Comprehensive structural connectivity features to enhance structural wiring modeling**

From the above, dMRI can be used to model the deep white matter fibers. However, modeling of superficial white matter and connections within gray matter are needed.

**Geodesic distance (GD)** can measure cortical spatial proximity, which is particularly useful for predicting short-range cortico-cortical connections and can be used to simulate lateral connections within gray matter (14). It may reflect the wiring cost, enabling estimation of short-range connections within gray matter and superficial white matter. Based on neuroanatomical data on cat, a research showed that the majority of cortico-cortical connections can be well predicted by distances between the cortical regions (21).

**Microstructural profile covariance (MPC)**：Within the gray matter, myelin sheaths envelop some neuronal axons. In recent years, the T1w/T2w ratio has shown to be sensitive to myelin content, thus serving as a proxy for microstructure in gray matter (22). The recently proposed microstructure profile covariance (MPC) method could be used to model cortico-cortical connections based on the similarity of microstructure profiles sampled along cortical columns across brain regions, which has indicated its biological basis (23).

The “structural model” proposed in the 1990s posited that cortical regions with similar cytoarchitecture are more likely to be connected. In recent years, evidence from animal experiments and neuroimaging have validated this theory. For instance, a recent study using tracers and cellular staining techniques in cat, mouse, and macaque monkeys demonstrated a correlation between cytoarchitectural similarity and connection probability (24). In humans, research utilizing the ultrahigh-resolution BigBrain dataset and HCP dMRI data suggests that cortical regions with higher similarity in laminar cytoarchitectonic patterns have a greater likelihood of being connected (25). Therefore, the MPC approach may estimate structural connectivity probability by evaluating the similarity of microstructure profiles between gray matter regions, based on the "structural model" hypothesis that the likelihood of connections is closely related to the microstructural similarity of cortical areas (23, 26).

Therefore, GD and MPC features can enhance the modeling of cortical wiring. Recently, the multiscale structural wiring model was introduced by combining connectivity features across micro and macro scale, encompassing diffusion MRI tractography, GD, and MPC (27), which could comprehensively capture the complex connectivity characteristics of the brain.

（4）**Neurobiological validation of the multiscale structural wiring model**

The neurobiological validity of the model has been demonstrated through a series of validation experiments (27). These experiments showed that the new wiring space reflects cortical microcircuit features, including pyramidal neuron depth and glial expression. Furthermore, the model enabled effective simulations of functional connectivity and dynamics based on resting-state functional magnetic resonance imaging (rs-fMRI) and human intracranial electroencephalography (EEG) coherence.

Although we assessed additional analyses incorporating MSN, its biological implications were not sufficient to reflect the cortical wiring. To maintain the biological clarity of the cortical wiring model, we decided to retain the original three features. In addition to considerations related to cortical wiring model, another key reason for not incorporating MSN into our model is that the analysis content in the following section is the relationship between the multiscale structural gradient and macroscopic morphological networks and features, if included in MSN will cause data confusion. Thus, we chose to retain the original model for analysis in the main text.

Q2. The authors utilized workbench commands (-surface-geodesic-distance) to compute the distance of each pair of centroid vertices within the given parcel. However, given the limitation of this approach in calculating the geodesic distance (GD), only intrahemispheric distance. Calculating interhemispheric GD by averaging GD between the two hemispheres would introduce errors. A recently published GD calculation approach that combines surface and volume-based GD calculations. This approach ﬁrst matched each vertex to the nearest voxel in volume space. Then, the distance to all other voxels traveling through a gray/white matter mask was calculated using a Chamfer propagation. I strongly recommend that authors use this method to calculate geometric distance matrixes.

**R**: Thank you for your valuable suggestion regarding the calculation of geodesic distance (GD). We appreciate your recommendation to use a more comprehensive approach that combines surface and volume-based GD calculations. Following your advice, we have implemented the suggested method, which involves matching vertices to the nearest voxels in volume space and calculating distances through a gray/white matter mask using Chamfer propagation. We have subsequently re-run all analyses using this improved GD calculation method.

As shown in the Fig 1, after adopting the new method, the first gradient remained similar to the previous pattern. However, the second gradient became a left-right brain gradient, which may be attributed to the recently published GD calculation approach's ability to calculate inter-hemispheric geodesic distances, thereby introducing inter-hemispheric information. The third gradient resembled the previous second gradient, approximating an anterior-posterior pattern. Therefore, our study focused on the first and third gradients. Moreover, based on the updated results, our conclusions remained largely consistent with our previous findings.

We made the following modifications to our methods:

*“Based on the mid-thickness surface of the individual native surface, the GD was calculated as the shortest distance between two nodes along the surface. Consistent with the previous study, we first calculated the Euclidean distances from all vertices to each other and identified the vertex with the minimum total distance, designated as the centroid vertex for each parcel (27). Next, we matched each centroid vertex to its nearest voxel in volumetric space. We then applied Chamfer propagation using the imGeodesics Toolbox (https://github.com/mattools/matImage/wiki/imGeodesics) to calculate distances to all other voxels while traversing through a grey/white matter mask(39). This method enables the estimation of inter-hemispheric projections (27), unlike previous approaches that focused solely on intra-hemispheric distances (14, 35). Finally, we projected the distance estimations back from volumetric to surface space, averaged them within each node, and created a symmetric distance matrix.* ***[Line 763-774]***

”

\

**Fig 1.** **Multiscale structural gradients during childhood and adolescence. (A)** The matrices containing the structural features of geodesic distance, microstructural profile covariance, and diffusion MRI tractography were concatenated and transformed into an affinity matrix, followed by the diffusion map embedding algorithm. The first three gradients capture the largest proportion of the variance. The group-averaged gradients were projected onto the cortical surface and visually represented (right). (**B)** The global density map of the principal gradient for six age-specific groups. (**C)** Radar plot of the principal gradient for comparison between the 6–7-year-old group and other age-specific groups based on Yeo functional networks (left) (40) and laminar differentiation parcellation (right) (41). **(D)** The first and third structural gradients mapped into a 2D gradient space for the 6–7-, 9-, and 12–13-year-old groups.

Q3. The authors performed PCA to generate a concise representation of the morphometric features (5 cortical morphometric features, CT, GMV, SA, MC, and GC). I found it bizarre that the physical significance of morphometric PC1.
Previous studies demonstrated that the principal gradient of MSN reveals diverged hierarchical organization in sensory-motor cortices (Yang Siqi, Cell Reports, 2021; Li Jiao, et al. Plos Biology, 2024). I am interested in the relationships between MSN gradient changes and multi-scale structure gradient changes from childhood to adolescence.
**R:** We thank the reviewer for the insightful comment and suggestions. We appreciate the opportunity to clarify our approach and provide additional analyses.

Regarding the use of PCA on the five cortical morphometric features (CT, GMV, SA, MC, and GC), we employed this method to create a concise representation due to the similar spatial patterns observed among these features. The first principal component (PC1) captures an overview of the morphometric features. However, to ensure a comprehensive analysis, we also examined each feature individually to identify which specific morphological characteristics correlate with the development of the multiscale structural gradient.

We appreciate your suggestion to investigate the relationship between MSN gradients and multi-scale structural gradient changes. Following your recommendation, we conducted additional analyses:

1. We constructed morphometric similarity networks (MSN) for each individual based on the five morphometric features.
2. We performed a correlation analysis between the edges of MSN and the edges of multiscale structural connectivity, which revealed a significant correlation. This finding suggests that brain regions with structural connections also tend to have similar macroscopic morphological features.
3. We calculated MSN gradients and found that the spatial patterns and developmental effects of the first MSN gradient correlated with those of multiscale structural gradient 1. This result aligns with our conclusion from the PCA method, further supporting the notion that the development of the multiscale structural gradient is coordinated with the development of macroscopic morphological features.

These additional MSN analyses have been included as S9 Fig to provide a more comprehensive understanding of the relationship between morphometric features and multiscale structural gradients. We revised the manuscript as follows:

*“Based on the five morphometric features, we constructed morphometric similarity networks (MSN) for each subject (S9 Fig). There is a significant correlation between the edges of MSN and the edges of multiscale structural connectivity (S9 Fig B). Furthermore, MSN principal gradient is also significantly correlated with multiscale structural gradient 1(r=-0.65, p_surrogate_ =0.00) (S9 Fig C). The ΔAdj R^2^-maps of multiscale structural gradient 1 and MSN gradient 1 also exhibited a significant spatial correlation (r=-0.16, p_surrogate_ <0.01) (S9 Fig D).*” ***[Line 329-334]***

**S9 Fig. Association between the multiscale structural principal gradient and MSN gradient. (A)** The MSN was fed into diffusion map embedding algorithm. The first gradient captures the largest proportion of the variance. The group-averaged gradients were projected onto the cortical surface and visually represented (right). **(B)** The correlation between MSN edges and multiscale structural connectivity edges. **(C)** Spatial correlation between the multiscale structural principal gradient and MSN gradient 1. Each dot represents a brain region. The significance level was corrected for spatial autocorrelation (p _surrogate_=0.00). **(D)** Spatial correlation between age-related ΔAdj R^2^-maps of the multiscale structural principal gradient and MSN gradient 1 (p _surrogate_=0.00). **(E)** The spatial pattern of age-effect on the principal gradient, with the age effect quantified using ΔAdj R^2^. The results that survived Bonferroni correction are circled by black lines (Bonferroni corrected p<0.05).

Q4. The authors investigated multiscale structure-function coupling during youth, which was calculated as the Spearman rank correlation between structural connectivity and FC profiles at the nodal level. Why do the authors not investigate the relationship between functional connectivity and multiscale cortical structural differentiation (i.e., the Euclidean distance in the wiring space)? It would be interesting to see the longitudinal structure-function coupling between age effects on functional connectivity and structural differentiation.
**R:** We appreciate the reviewer’s suggestion to investigate the relationship between functional connectivity and multiscale cortical structural differentiation using the Euclidean distance in the wiring space. We have conducted additional analyses following the method described in (42) to address this comment.

Specifically, we constructed a wiring space based on the first and third multiscale gradients and calculated Euclidean distances between nodes as a measure of multiscale structural differentiation. We then computed average differentiation and functional connectivity within and between Yeo functional systems.

Our analysis revealed a negative correlation between group-average structural differentiation and functional connectivity at the network level (r=-0.53, p=0.01), indicating that more differentiated systems showed weaker functional connectivity.

To examine developmental effects, we used GAMMs with FWE correction and found that most inter-system structural differentiation increased with age. We then correlated these age-related increases in differentiation with functional connectivity developmental effects. Consistent with the findings reported by (42) which focused on 14-25 year old population, our analysis showed a negative correlation (r=-0.26, p=0.24). This finding showed that greater increase in structural differentiation during development corresponds to the weaker increase in functional connectivity, indicating the interaction between multiscale structural and functional organization.

These additional analyses have been included as S13 Fig to provide further insights into the relationship between multiscale structural differentiation and functional connectivity during development, complementing our original multiscale structure-function coupling investigation.

**S13 Fig.** **Association between functional connectivity and structural differentiation. (A, B)** The functional connectivity matrix/multiscale structural differentiation matrix was summarized according to Yeo functional networks (40). **(C)** Correlation between multiscale structural differentiation and functional connectivity. **(D, E)** Age-effect on functional connectivity and multiscale structural differentiation, with the age-effect quantified using ΔAdj R^2^ and a significant network reported with an asterisk (Bonferroni corrected p<0.05). **(F)** The correlation between age effects on multiscale structural differentiation and age effects on functional connectivity.

Q5. To quantify the effect of age on multiscale structural gradients during development, the authors constructed linear mixed-effect (LME) models. However, the development process is not always linear (Bethlehem Richard AI, et al. Nature 2022). A generalized additive model for location, scale, and shape (GALSS), a robust method for modeling nonlinear growth trajectories, would be useful for constructing the development process from childhood to adolescence.

**R:** We thank the reviewer for this insightful feedback regarding the modeling of nonlinear developmental processes during development. While we appreciate the merits of using GAMLSS to accommodate a wide range of data distributions and complex nonlinear relationships, we chose to use a generalized additive mixed model (GAMM) in our study's context. GAMLSS can accommodate various data distributions which offers advantages in handling non-normal distributions. Since our data was obtained from a single center, and has been verified to follow a normal distribution, we opted to use a generalized additive mixed model (GAMM) with penalized splines in R (version 4.3.0) (43) utilizing the mgcv package(44) to explore the relationship between brain variables and age. This model incorporated age as a smooth term, subject ID as a random effect, and sex and mFD as linear covariates. We employed cubic splines for the smooth term, offering computational efficiency and appropriateness for data sparsity at boundaries. The restricted maximum likelihood (REML) approach was used for the optimization of smoothing parameters(45). The GAMM smooth term for age produces a spline, modeled by weighted basis functions, that represents a developmental trajectory for each metric. To prevent overfitting of the spline, the maximum basis complexity (k) was set to 3, as the Bayesian information criterion (BIC) comparison indicated lower BIC values for most global gradient measures at k = 3 rather than k = 4. We assessed the effect size of each age spline by calculating the change in adjusted R² (ΔAdj R²) between the full GAMM model and a reduced model excluding the age term. Statistical significance was evaluated using analysis of variance (ANOVA) to compare the full and reduced models. Since ΔAdj R² is an effect size and directionless, we extracted the sign of the age coefficient from the equivalent linear model and assigned it to ΔAdj R².

This model was utilized to characterize developmental effects for global gradient measures and regional gradient scores, and we further plotted the developmental trajectory of the principal gradient for each region. Additionally, throughout the manuscript, we employed the GAMM model, including for analyses of morphological and structure-function coupling sections. For the HCP-D cross-sectional dataset used for validation, we removed the random effect term, keeping the rest unchanged.

We believe that the GAMM approach provides a robust framework for modeling linear and nonlinear developmental effects in our study's context.

Q6. The physical meaning of t-statistic of age-related changes is difficult to comprehend. To provide insight into the overall magnitude and direction of regional age effects, many previous studies (such as Sydnor Valerie, et al. Nature neuroscience, 2023; Li Jiao, et al. Plos Biology, 2024) have represented the age effects (R2 values) as the age-related regional changes during development. Therefore, I suggest replacing the t-statistic with the age effect (R2 values).

**R:** We thank the reviewer for this valuable suggestion. We have already addressed this concern by replacing the linear mixed-effects models with GAMM models and using ΔAdj R² values instead of t-statistics to represent effect sizes.

Q7. It would be necessary to use an independent data set (such as., HCPD, aged 6 to 14 years) to verify the repeatability of the results.
**R:** We appreciate the reviewer's suggestion regarding independent verification of our results. We have already utilized the Human Connectome Project Development (HCP-D) dataset, which includes participants aged 6-14 years, to validate our main findings. This age range aligns closely with our original study population. The HCP-D dataset provides multi-modal neuroimaging data, including T1w, T2w, dMRI, and rs-fMRI, allowing for a comprehensive validation of our results across multiple imaging modalities. We conducted analyses on the HCP-D dataset that parallel our original study. We first characterized the developmental patterns of multiscale structural gradients during childhood and adolescence at whole brain level and regional level (S5 Fig). Furthermore, we explored the associations between the principal gradient and the cortical morphology as well as their development interaction patterns (S8 Fig). We also examined the associations between development of multiscale structure–function coupling and the change of participation coefficient (S10 Fig). Finally, we explored the relationship between cognitive scores and the multiscale structural principal gradient across individuals via partial least squares correlation (PLSC) analysis (S11 Fig). These results obtained from this independent dataset are consistent with our original findings, thus providing additional support for the robustness of our conclusions.

We believe this approach addresses the reviewer's concern for independent verification while also strengthening the overall reliability of our results. The details of these validation analyses using the HCP-D dataset have been added to the supplementary materials of our manuscript.

**S5 Fig. Independent replication of multiscale structural gradients during childhood and adolescence based on Schaefer 400 atlas.** The same steps were conducted to regenerate the multiscale structural gradients using an independent HCP-D dataset aged 6-14 years old.

**S8 Fig. Independent replication of association between the multiscale structural principal gradient and morphometric features.** Results of the analysis of relationships between cortical morphological features using the independent HCP-D dataset for ages 6-14, employing the same steps as in the discovery dataset.

**S10 Fig. Independent replication of multiscale structure–function coupling during development.** Results of the analysis of multiscale structure–function coupling using the independent HCP-D dataset for ages 6-14, employing the same steps as in the discovery dataset.

**S11 Fig. Independent replication of PLSC analysis between the principal gradient and cognitive scores in HCD-D dataset.** (A) Pearson correlations between the composite scores of the principal gradient and composite scores of cognitive measures. The inset figure shows the null distribution of singular values estimated by the permutation test (n = 1000). (B) Loadings of cognitive measures were calculated by Pearson correlation between the cognitive measurements and their composite scores. The shadows represent significant loadings tested by bootstrap resampling (n=1000). Cognitive measures included fluid intelligence, episodic memory, executive function/cognitive flexibility, inhibition, language/reading decoding, vocabulary comprehension, processing speed, and working memory (C) Gradient loadings were calculated by Pearson correlation between gradient 1 and their composite scores. The loadings of regions with black lines were subjected to a significance test by bootstrap resampling (n=1000).

Q8. Given that right hemisphere data were only available from 2 donors, the authors opted to utilize the data from the left hemisphere for our analysis. Using the default parameters, a 416 ×15631 matrix was obtained. Why did the authors obtain a 416 ×15631 gene expression matrix instead of a 500 ×15631 matrix.
**R:** We thank the reviewer for this critical comment. In our analysis, regional microarray expression data were processed with the abagen toolbox (version 0.1.3; https://github.com/rmarkello/abagen) using a 1000-region volumetric atlas in MNI space (46). The discrepancy in the dimensions of the gene expression matrix (416 × 15631 instead of the expected 500 × 15631) results from several data processing steps and constraints we applied:

1. **Probe reannotation and filtering**: During the initial processing, probes without a valid Entrez ID were discarded, as well as those with intensity less than background noise in ≥50% of samples. This filtering step inherently reduces the number of viable probes and subsequently impacts the number of regions that can be fully represented.
2. **Sample assignment constraints**: Samples were assigned to brain regions based on their MNI coordinates, with strict constraints on assignment: samples had to be within 2 mm of a parcel and were constrained by hemisphere and structural division (e.g., cortex, subcortex). We used a 1000-region volumetric atlas in MNI space. Only samples successfully matched to these regions were included, and any unassigned samples due to these criteria were discarded.

Follow these method steps and we finally get the gene expression matrix of 416 × 15631.We have revised the manuscript as:

*“Using the default parameters, we finally obtained a 416 ×15631 (region × gene) matrix. To be more specific, the microarray probes were reannotated using according to data provided by (119), and probes not matched to a valid Entrez ID were discarded. Probes whose expression intensity exceeded background noise in 50% of samples across all donors were retained (120). For genes with multiple probes, the probe with the most consistent pattern of regional variation across six donors was selected as representative probe, resulting in 15,631 genes. Then samples were assigned to brain regions based on their MNI coordinates, with strict constraints on assignment: samples had to be within 2 mm of a parcel and were constrained by hemisphere and structural division. We used Schaefer 1000 volumetric atlas in MNI space. within-donor normalization across donors were performed to mitigate donor-specific effects. Finally, samples were averaged first within regions for each donor, then across donors to create the regional expression matrix. Given that right hemisphere data were only available from 2 donors, we opted to utilize the data from the left hemisphere for our analysis.”*

Q9. Abstract is somewhat redundant. Authors should simplify and improve abstract.

**R:** We thank the reviewer for this point. We revised the abstract as:

*“From childhood to adolescence, the structural organization of the human brain undergoes dynamic and regionally heterogeneous changes across multiple scales, from synapses to macroscale white matter pathways. However, during this period, the developmental process of multiscale structural architecture, its association with cortical morphological changes, and its role in the maturation of functional organization remain largely unknown. Here, using two independent multimodal imaging developmental datasets aged 6 to 14 years, we investigated developmental process of multiscale cortical organization by constructing an in vivo multiscale structural connectome model incorporating white matter tractography, cortico–cortical proximity, and microstructural similarity. By employing the gradient mapping method, the principal gradient derived from the multiscale structural connectome effectively recapitulated the sensory-association axis. Our findings revealed a continuous expansion of the multiscale structural gradient space during development, characterized by enhanced differentiation between primary sensory and higher-order transmodal regions along the principal gradient. This age-related differentiation paralleled regionally heterogeneous changes in cortical morphology. Furthermore, the developmental changes in coupling between multiscale structural and functional connectivity were correlated with functional specialization refinement, as evidenced by changes in the participation coefficient. Notably, the differentiation of the principal multiscale structural gradient was associated with improved cognitive abilities, such as enhanced working memory and attention performance, and potentially underpinned by synaptic and hormone-related biological process. These findings advance our understanding of the intricate maturation process of brain structural organization and its implications for cognitive performance.”*

Q10. The full title of the manuscript is too long, and a short title is strongly recommended.
**R:** We thank the reviewer for this point. We revised the title as:

*“Multiscale structural gradient differentiation correlates with cortical morphology maturation and functional specialization from childhood to adolescence”.*

Q11. The Results section contains too much description of the Methods. The authors should mainly describe the results and what they reveal.

**R:** We thank the reviewer for this comment regarding the Results section. We have thoroughly revised the Results section to be more concise and results-focused, removing excessive methodological details. The methodological information has been appropriately relocated to the Methods section.

**References**

1.	Maier-Hein KH, Neher PF, Houde J-C, Côté M-A, Garyfallidis E, Zhong J, et al. The challenge of mapping the human connectome based on diffusion tractography. Nature communications. 2017;8(1):1349.

2.	Jones DK, Knösche TR, Turner R. White matter integrity, fiber count, and other fallacies: the do's and don'ts of diffusion MRI. Neuroimage. 2013;73:239-54.

3.	Dhollander T, Connelly A, editors. A novel iterative approach to reap the benefits of multi-tissue CSD from just single-shell (+ b= 0) diffusion MRI data. Proc ISMRM; 2016.

4.	Jeurissen B, Tournier J-D, Dhollander T, Connelly A, Sijbers J. Multi-tissue constrained spherical deconvolution for improved analysis of multi-shell diffusion MRI data. NeuroImage. 2014;103:411-26.

5.	Smith RE, Tournier J-D, Calamante F, Connelly A. Anatomically-constrained tractography: improved diffusion MRI streamlines tractography through effective use of anatomical information. Neuroimage. 2012;62(3):1924-38.

6.	Smith RE, Tournier J-D, Calamante F, Connelly A. SIFT: Spherical-deconvolution informed filtering of tractograms. NeuroImage. 2013;67:298-312.

7.	Budd JML, Kisvárday ZF. Communication and wiring in the cortical connectome. Frontiers in Neuroanatomy. 2012;6.

8.	Salin PA, Bullier J. Corticocortical connections in the visual system: structure and function. Physiological Reviews. 1995;75(1):107-54.

9.	Schüz A, Miller R. Cortical areas : unity and diversity. London ;: Taylor & Francis; 2002.

10.	Sara L, Reinder VDW, Hong SJ, Casey P, Shahin T, Lowe AJ, et al. Multiscale Structure-Function Gradients in the Neonatal Connectome. Cerebral Cortex. 2019(1):1.

11.	Rockland KS. Elements of Cortical Architecture. Cerebral Cortex: Springer US; 1997. p. 243-93.

12.	Schwartz ML, Goldman-Rakic PS. Single cortical neurones have axon collaterals to ipsilateral and contralateral cortex in fetal and adult primates. Nature. 1982;299(5879):154-5.

13.	Bullier J, Kennedy H, Salinger W. Branching and laminar origin of projections between visual cortical areas in the cat. Journal of Comparative Neurology. 1984;228(3):329-41.

14.	Ecker C, Ronan L, Feng Y, Daly E, Murphy C, Ginestet CE, et al. Intrinsic gray-matter connectivity of the brain in adults with autism spectrum disorder. Proceedings of the National Academy of Sciences. 2013;110(32):13222-7.

15.	Dorkenwald S, Turner NL, Macrina T, Lee K, Lu R, Wu J, et al. Binary and analog variation of synapses between cortical pyramidal neurons. Elife. 2022;11:e76120.

16.	Reveley C, Seth AK, Pierpaoli C, Silva AC, Yu D, Saunders RC, et al. Superficial white matter fiber systems impede detection of long-range cortical connections in diffusion MR tractography. Proceedings of the National Academy of Sciences. 2015;112(21):E2820-E8.

17.	Zhang F, Daducci A, He Y, Schiavi S, Seguin C, Smith RE, et al. Quantitative mapping of the brain’s structural connectivity using diffusion MRI tractography: A review. Neuroimage. 2022;249:118870.

18.	Guevara M, Guevara P, Román C, Mangin J-F. Superficial white matter: A review on the dMRI analysis methods and applications. NeuroImage. 2020;212:116673.

19.	Kirilina E, Helbling S, Morawski M, Pine K, Reimann K, Jankuhn S, et al. Superficial white matter imaging: Contrast mechanisms and whole-brain in vivo mapping. Science Advances. 2020;6(41):eaaz9281.

20.	Conturo TE, Lori NF, Cull TS, Akbudak E, Snyder AZ, Shimony JS, et al. Tracking neuronal fiber pathways in the living human brain. Proceedings of the National Academy of Sciences. 1999;96(18):10422-7.

21.	Scannell JW, Blakemore C, Young MP. Analysis of connectivity in the cat cerebral cortex. Journal of Neuroscience. 1995;15(2):1463-83.

22.	Glasser MF, Van Essen DC. Mapping human cortical areas in vivo based on myelin content as revealed by T1-and T2-weighted MRI. Journal of neuroscience. 2011;31(32):11597-616.

23.	Paquola C, Vos De Wael R, Wagstyl K, Bethlehem RAI, Hong S-J, Seidlitz J, et al. Microstructural and functional gradients are increasingly dissociated in transmodal cortices. PLoS biology. 2019;17(5):e3000284.

24.	Goulas A, Majka P, Rosa MGP, Hilgetag CC. A blueprint of mammalian cortical connectomes. PLoS biology. 2019;17(3):e2005346.

25.	Wei Y, Scholtens LH, Turk E, Van Den Heuvel MP. Multiscale examination of cytoarchitectonic similarity and human brain connectivity. Network Neuroscience. 2018;3(1):124-37.

26.	García-Cabezas MÁ, Zikopoulos B, Barbas H. The Structural Model: a theory linking connections, plasticity, pathology, development and evolution of the cerebral cortex. Brain Structure and Function. 2019;224(3):985-1008.

27.	Paquola C, Seidlitz J, Benkarim O, Royer J, Klimes P, Bethlehem RAI, et al. A multi-scale cortical wiring space links cellular architecture and functional dynamics in the human brain. PLoS Biology. 2020;18(11).

28.	Burt JB, Helmer M, Shinn M, Anticevic A, Murray JD. Generative modeling of brain maps with spatial autocorrelation. NeuroImage. 2020;220:117038.

29.	Sydnor VJ, Larsen B, Bassett DS, Alexander-Bloch A, Fair DA, Liston C, et al. Neurodevelopment of the association cortices: Patterns, mechanisms, and implications for psychopathology. Neuron. 2021;109(18):2820-46.

30.	Baum GL, Cui Z, Roalf DR, Ciric R, Betzel RF, Larsen B, et al. Development of structure–function coupling in human brain networks during youth. Proceedings of the National Academy of Sciences. 2020;117(1):771-8.

31.	Marek S, Tervo-Clemmens B, Calabro FJ, Montez DF, Kay BP, Hatoum AS, et al. Reproducible brain-wide association studies require thousands of individuals. Nature. 2022;603(7902):654-60.

32.	Helmer M, Warrington S, Mohammadi-Nejad A-R, Ji JL, Howell A, Rosand B, et al. On the stability of canonical correlation analysis and partial least squares with application to brain-behavior associations. Communications Biology. 2024;7(1):217.

33.	Fulcher BD, Arnatkeviciute A, Fornito A. Overcoming false-positive gene-category enrichment in the analysis of spatially resolved transcriptomic brain atlas data. Nature communications. 2021;12(1):2669.

34.	Burt JB, Demirtaş M, Eckner WJ, Navejar NM, Ji JL, Martin WJ, et al. Hierarchy of transcriptomic specialization across human cortex captured by structural neuroimaging topography. Nature neuroscience. 2018;21(9):1251-9.

35.	Margulies DS, Ghosh SS, Goulas A, Falkiewicz M, Huntenburg JM, Langs G, et al. Situating the default-mode network along a principal gradient of macroscale cortical organization. Proceedings of the National Academy of Sciences. 2016;113(44):12574-9.

36.	Xu T, Nenning K-H, Schwartz E, Hong S-J, Vogelstein JT, Goulas A, et al. Cross-species functional alignment reveals evolutionary hierarchy within the connectome. Neuroimage. 2020;223:117346.

37.	Seidlitz J, Váša F, Shinn M, Romero-Garcia R, Whitaker KJ, Vértes PE, et al. Morphometric similarity networks detect microscale cortical organization and predict inter-individual cognitive variation. Neuron. 2018;97(1):231-47.

38.	Wang J, He Y. Toward individualized connectomes of brain morphology. Trends in Neurosciences. 2024.

39.	Larivière S, Weng Y, Vos de Wael R, Royer J, Frauscher B, Wang Z, et al. Functional connectome contractions in temporal lobe epilepsy: Microstructural underpinnings and predictors of surgical outcome. Epilepsia. 2020;61(6):1221-33.

40.	Yeo BT, Krienen FM, Sepulcre J, Sabuncu MR, Lashkari D, Hollinshead M, et al. The organization of the human cerebral cortex estimated by intrinsic functional connectivity. J Neurophysiol. 2011;106(3):1125-65.

41.	Mesulam MM. Behavioral Neuroanatomy: Large-Scale Networks, Association Cortex, Frontal Syndromes, the Limbic System, and Hemispheric Specializations. United States: Oxford University Press, Incorporated; 2000.

42.	Park B-y, Paquola C, Bethlehem RAI, Benkarim O, Neuroscience in Psychiatry Network C, Mišić B, et al. Adolescent development of multiscale structural wiring and functional interactions in the human connectome. Proceedings of the National Academy of Sciences. 2022;119(27):e2116673119.

43.	Team RC. R: A language and environment for statistical computing. Foundation for Statistical Computing, Vienna, Austria. 2013.

44.	Wood SN. mgcv: GAMs and generalized ridge regression for R. R news. 2001;1(2):20-5.

45.	Wood SN. Generalized additive models: an introduction with R: chapman and hall/CRC; 2017.

46.	Arnatkevic̆iūtė A, Fulcher BD, Fornito A. A practical guide to linking brain-wide gene expression and neuroimaging data. NeuroImage. 2019;189:353-67.
