# Supplementary material for: The multiscale brain structural re-organization that occurs from childhood to adolescence correlates with cortical morphology maturation and functional specialization
Source: PLoS Biol. 2025 Apr 1;23(4):e3002710. doi: 10.1371/journal.pbio.3002710 (PMC12017512; doi:10.1371/journal.pbio.3002710)
Supplement: Attachment — Submitted filename: Response_to_Reviewers_auresp_3.docx [file pbio.3002710.s020.docx]

**Point-by-point response to Reviewer: 1**
The authors have address most of my comments. However, the new Fig S1 indicates that their model is mainly determined by MPC.

Since the MPC, TS, and GD maps are all correlated to some extent, the authors should examine partial correlations.

If it turns out that their model is largely driven by MPC, then the authors should demonstrate the benefits of their chosen model over just using MPC measures.

**R:** We thank the reviewer for raising this insightful comment. In response, considering the collinearity among individual feature (MPC, TS, GD) gradient maps, we re-calculated partial correlations between the multiscale structural gradients and individual feature gradients, while controlling for the influence of the other features, to assess the relative contributions of MPC, TS, and GD in our model. As shown in S1 Fig, both MPC and GD gradient maps have significant spatial correlations with the multiscale structural principal gradient after controlling for other predictors, with MPC exhibiting stronger correlations (r=0.97, p_surrogate_=0.00) compared to GD (r=0.36, p_surrogate_=0.00).

**S1 Fig. Correlations between individual feature gradients (MPC, TS, GD) and multiscale structural gradients.** The x-axis represents the top three gradients of MPC, TS, and GD, while the y-axis represents the partial correlations with multiscale structural gradients. The top, middle, and bottom plots correspond to the top three multiscale structural gradients, respectively. Red dots indicate actual correlation coefficients. Spatial autocorrelation was corrected using 1000 surrogate maps generated via the variogram matching approach (55). Box plots and density plots show the null distribution of 1000 correlation coefficients.

Although the above results indicate that the multiscale structural gradient is primarily driven by MPC and GD, we maintain that retaining all three features is essential for modeling multiscale structural connectivity. Beyond the theoretical foundations of the multiscale structural wiring model addressed in our previous response to reviewers, we evaluated the model's validity from two perspectives to further justify the benefits of using the three-feature model over a reliance on MPC alone.

(a) **Predicting functional connectivity using multiple structural connectivity features**

Intricate connection patterns among neural elements form a complex hierarchical network that promotes signaling and molecular transport in the brain (1). Complex networks of synapses enable signaling between neurons, forming neural circuits (2). The validity of a structural connectivity model can be assessed by its ability to predict functional connectivity (FC) - if the model successfully captures functional interactions, it demonstrates that the underlying structural features are indeed meaningful determinants of brain function. Here, we leveraged structural connectivity features to predict FC patterns, aiming to validate the structural model through its predictive performance.

Based on group-averaged FC and structural connectivity matrices, we used different combinations of structural connectivity features to predict region-level FC via multiple linear regression models. For the prediction of FC, we employed seven different combinations of structural connectivity features (MPC+GD+TS; GD+TS; MPC+TS; MPC+GD; MPC; GD; TS). For each brain region, we used its structural connectivity profile with the whole brain as predictors to predict its functional connectivity profile with the whole brain. This resulted in one fitted value per brain region, with adjusted R² used to measure the goodness of fit of the model. As shown in Figure 1, the vertical axis represents different combinations of features, while the horizontal axis displays the distribution of adjusted R² values for predicting regional-level functional connectivity along with their corresponding boxplots. The scatter points represent adjusted R² values of different brain regions. We conducted two-sample t-tests to compare the predictions from the MPC+GD+TS model with those from other models, and significant differences are indicated with an asterisk (*) (p<0.05). Notably, among seven different combinations, the three-feature combination (MPC+TS+GD) significantly outperformed all other combinations. Among the predictors, GD was the strongest predictor, followed by TS, and then MPC. Importantly, removing any one of the features significantly degraded model performance, underscoring the critical role of all three features in explaining FC. Thus, the combined model provides a more comprehensive and accurate reflection of macroscopic functional interactions, offering deeper insights into the structure-function relationship in the brain.

**Figure 1**. The distribution of adjusted R² values for predicting node-level functional connectivity (FC) across different feature combinations. The vertical axis represents the various combinations of features (MPC, GD, TS), while the horizontal axis shows the adjusted R² values along with corresponding boxplots. Each scatter point represents a different brain region. Two-sample t-tests were performed to compare the predictions from the MPC+GD+TS model with those from other models, and significant differences are indicated with an asterisk (*) (p<0.05).

(b) **Predicting gene similarity network using multiple structural connectivity features**

Brain development follows precisely orchestrated cellular and molecular processes governed by genetic mechanisms (3). Previous study based on anterograde tract-tracing data have found greater transcriptomic similarity or gene co-expression between regions of the mouse brain were more likely to be axonally connected (4). This observation led to the hypothesis that brain regions sharing similar gene expression profiles would possess comparable structural properties and demonstrate increased probability of structural connectivity (5). The correspondence between structural connectivity patterns and gene expression similarity networks would provide evidence for the biological validity of structural connectivity modeling. Thus, we used structural connectivity features to predict gene similarity network derived from Allen Human Brain Atlas to further validate the biological foundation.

Specifically, we constructed a 416×416 gene similarity network by computing Pearson correlation coefficients between gene expression profiles for each pair of brain regions, using the gene expression matrix(416×15631; region × gene) obtained from the main text. In this network, edges represent the degree of similarity between regional gene expression profiles. Following the same methodology employed for predicting functional connectivity, we applied multiple linear regression models using seven combinations of structural connectivity features (MPC+GD+TS; GD+TS; MPC+TS; MPC+GD; MPC; GD; TS) to predict this gene similarity network. As shown in Figure 2, the three-feature combination achieved the best predictive fit for the gene similarity network. Removing any single feature again resulted in declines in prediction performance, further highlighting the synergistic utility of MPC, TS, and GD.

**Figure 2**. The distribution of adjusted R² values for predicting node-level gene similarity network across different feature combinations. The vertical axis represents the various combinations of features (MPC, GD, TS), while the horizontal axis shows the adjusted R² values along with corresponding boxplots. Each scatter point represents a different brain region. Two-sample t-tests were performed to compare the predictions from the MPC+GD+TS model with those from other models, and significant differences are indicated with an asterisk (*) (p<0.05).

Together, these results emphasize the robustness and utility of the full three-feature model, demonstrating that it consistently outperforms models using only MPC or other subsets of features in predicting both functional connectivity and gene similarity. While MPC is a contributing factor, GD and TS each offer independent and important predictive power, making the three-feature model a significantly stronger and biologically meaningful model to capture complex brain organization.

**Point-by-point response to Reviewer: 2**
I thank the authors for their revisions. I only have few outstanding comments.

Q1. Please be more explicit in discussing limitations of using AHBA in the current work. Specifically, provide additional context that this analysis is a cross-dataset correlational inference, where patterns seen in typically developing cohorts are spatially associated to gene expression patterns seen in 6 left hemispheres and 2 right hemispheres of primarily adult donors. Please discuss some potential imitations of microarray gene expression analysis relative to eg RNASeq based approaches. Please emphasize the well known issue that correlation does not implicate causality necessarily.
**R:** Thank you for your constructive feedback. We appreciate the opportunity to clarify these issues in the revised manuscript. We have revised our discussion to explicitly address the limitations you have raised:

*“Third, the gene-related analysis was a cross-dataset correlational inference, where gene expression profiles were derived from a small sample of adult postmortem brains (6 left hemispheres and 2 right hemispheres), potentially limiting its generalizability, especially to developmental stages. Furthermore, the use of microarray-based gene expression may have lower sensitivity and accuracy compared to RNA-Seq, which offers greater transcriptomic coverage and precision (88).* *Future studies should incorporate pediatric-specific gene expression datasets with spatial resolution comparable to that of the AHBA, based on more precise transcriptomic measurement techniques. Notably, while we observe spatial correlations between typical cohort patterns and AHBA-derived gene expression, we emphasize that correlation does not imply causation. These limitations should be considered when interpreting our results.” 【Line 651-661】*

Q2. As for my Q6, I would think PLOS guidelines mandate the open access of data that are used for the main analysis, so the authors would at minimum need to upload the used feature data to eg off so that other groups can replicate results.

**R:** Thank you for highlighting this important point. We sincerely acknowledge the importance of data accessibility and appreciate your emphasis on ensuring transparency and reproducibility in scientific research. As this is a longitudinal study with ongoing follow-up data collection, we are currently unable to release the full dataset. However, the Principal Investigator plans to share the complete dataset, including all follow-up data, once the data collection and processing are finalized.

In the meantime, in line with the reviewer’s suggestion, we have uploaded the data used in the main analysis, including the feature data (MPC, TS, and GD) and cortical morphological data to a public repository on Zenodo ([https://zenodo.org/records/14440214](mailto:https://zenodo.org/records/14440214)). This will allow other researchers to replicate our results.

**References**

1.	Sporns O, Chialvo D, Kaiser M, Hilgetag CJTics. Organization, development and function of complex brain networks. Trends Cogn Sci. 2004;8(9):418-25.

2.	Douglas RJ, Martin KAC. Neuronal circuits of the neocortex. Annu Rev Neurosci. 2004;27(1):419-51.

3.	Silbereis JC, Pochareddy S, Zhu Y, Li M, Sestan N. The cellular and molecular landscapes of the developing human central nervous system. Neuron. 2016;89(2):248-68.

4.	Fulcher BD, Fornito A. A transcriptional signature of hub connectivity in the mouse connectome. Proceedings of the National Academy of Sciences. 2016;113(5):1435-40.

5.	Seidlitz J, Váša F, Shinn M, Romero-Garcia R, Whitaker KJ, Vértes PE, et al. Morphometric similarity networks detect microscale cortical organization and predict inter-individual cognitive variation. Neuron. 2018;97(1):231-47.
